# Phenolics Isolated from *Aframomum meleguta* Enhance Proliferation and Ossification Markers in Bone Cells

**DOI:** 10.3390/molecules22091467

**Published:** 2017-09-04

**Authors:** Ashraf B. Abdel-Naim, Abdullah A. Alghamdi, Mardi M. Algandaby, Fahad A. Al-Abbasi, Ahmed M. Al-Abd, Hossam M. Abdallah, Ali M. El-Halawany, Masao Hattori

**Affiliations:** 1 Medicinal Plants Research Unit, Deanship of Scientific Research, King Abdulaziz University, Jeddah 80230, Saudi Arabia; malgandaby@yahoo.com (M.M.A.); alabassif@hotmail.com (F.A.A.-A.); 2 Department of Pharmacology and Toxicology, Faculty of Pharmacy, King Abdulaziz University, Jeddah 21589, Saudi Arabia; ahmedmalabd@pharma.asu.edu.eg; 3 Department of Biochemistry, Faculty of Science, King Abdulaziz University, Jeddah 21523, Saudi Arabia; abdullah.elghamdi@gmail.com; 4 Department of Biological Sciences, Faculty of Science, King Abdulaziz University, Jeddah 21523, Saudi Arabia; 5 Pharmacology Department, Medical Division, National Research Centre, Giza 12622, Egypt; 6 Department of Natural Products, Faculty of Pharmacy, King Abdulaziz University, Jeddah 21589, Saudi Arabia; hmafifi2013@gmail.com; 7 Department of Pharmacognosy, Faculty of Pharmacy, Cairo University, Cairo 11562, Egypt; ahalawany2003@yahoo.com; 8 Institute of Natural Medicine, University of Toyama, Toyama 930-0194, Japan; cre421@yahoo.co.jp

**Keywords:** *Aframomum melegueta*, paradol, osteoporosis

## Abstract

Osteoporosis is a serious health problem characterized by decreased bone mineral density and deterioration of bone microarchitecture. Current antiosteoporotic agents exhibit a wide range of adverse effects; meanwhile, phytochemicals are effective and safer alternatives. In the current work, nine compounds belonging to hydroxyphenylalkane and diarylheptanoid groups were isolated from *Aframomum meleguea* seeds and identified as 6-gingerol (**1**), 6-paradol (**2**), 8-dehydrogingerdione (**3**), 8-gingerol (**4**), dihydro-6-paradol (**5**), dihydrogingerenone A (**6**), dihydrogingerenone C (**7**), 1,7-bis(3,4-dihydroxy-5-methoxyphenyl)heptane-3,5-diyl diacetate (**8**), and 1-(3,4-dihydroxy-5-methoxyphenyl)-7-(3,4-dihydroxyphenyl)heptane-3,5-diyl diacetate (**9**). The structures of isolated compounds were established by NMR and mass spectral data, in addition to referring to literature data. Exposure of MCF-7, MG-63, and SAOS-2 cells to subcytotoxic concentrations of the compounds under investigation resulted in accelerated proliferation. Among them, paradol was selected for further detailed biochemical analysis in SAOS-2 cells. DNA flowcytometric analysis of cell cycle distribution revealed that paradol did not induce any significant change in the proliferation index of SAOS-2 cells. Assessment of osteogenic gene expression revealed that paradol enhanced the expression of osteocyte and osteoblast-related genes and inhibited osteoclast and RUNX suppressor genes. Biochemically, paradol enhanced alkaline phosphatase activity and vitamin D content and decreased the osteoporotic marker acid phosphatase. In conclusion, paradol, which is a major constituents of *A. melegueta* seeds, exhibited potent proliferative and ossification characteristics in bone cells.

## 1. Introduction

Osteoporosis is a serious health problem that affects more than 200 million people worldwide [1]. Osteoporosis is characterized by a decrease in bone mineral density (BMD) and the deterioration of bone microarchitecture. It particularly affects postmenopausal women, thus leading to a significant loss of bone mineralized mass and leading to increased bone fragility and susceptibility to fracture [2]. The disease is sometimes described as a “silent disease”, as it is usually asymptomatic until a fracture ensues [3]. It causes significant morbidity, mortality, and a socioeconomic burden. In particular, osteoporosis-related hip fraction represents a heavy socioeconomic and medical burden. Unfortunately, worldwide hip fractures are expected to rise to 6.26 million in 2050 [4]. Post-menopausal women are particularly at a high risk, especially with the adoption of the Western sedentary lifestyle [5]. Postmenopausal osteoporosis is mainly managed with estrogen replacement therapy (ERT). This is in addition to bisphosphonates, selective estrogen receptor modulators (SERM), and calcitonin [6]. Unfortunately, the beneficial effects of these agents are counterbalanced by serious adverse events, including increased cancer and thromboembolic risks [7,8,9]. This necessitates the search for novel harmless antiosteoporotic drugs. In line, phytochemicals could win wide recognition among the public and scientific community [10]. Extensive efforts have been made to reveal the beneficial effects of plants on bone health. In this regard, several plants belonging to the *Zingiberaceae* family have shown promising properties that deserve additional investigation [11]. Turmeric, ginger, and alligator pepper (*Aframomum melegueta*) are examples of such plants. Curcumin, a diarylheptanoid obtained from turmeric, has been reported to suppress osteoclastogenesis and preserve the microarchitecture of bones [12,13]. Feeding pregnant rats with an aqueous extract of ginger resulted in fetuses with increased skeletal development [14]. Moreover, the hexane extract of ginger inhibited RANKL-induced osteoclastogenesis [15]. Gingerol significantly enhanced the proliferation, differentiation, and mineralization of osteoblast-like MG-63 cells [16]. *A. melegueta* K. Schum. (Zingiberaceae) is a common spice and medicinal herb abundantly found in Western and Central Africa [17]. Currently, it is grown in North Africa and Asia [18]. Phytochemically, hydroxyphenyl alkanes—such as gingerol and paradol and diarylheptanoids—represent the major constituents of *A. melegueta* seeds in addition to fatty acids and essential oils [19,20]. It has been used in folk medicine for stomachaches, diarrhea, hypertension, and tuberculosis [21,22]. El-Halawany et al. [18] reported that *A. melegueta* extract possesses a promising estrogen-modulating effect. However, the potential beneficial effect of *A. melegueta* and its active constituents on bone cells has not yet been examined. Therefore, the current study was designed to isolate, identify, and assess the activity of phytochemicals isolated from *A. melegueta* on bone cell proliferation and ossification markers.

## 2. Results

### 2.1. Isolation and Structural Identification of Major Phenolics from A. melegueta

**Figure 1:**
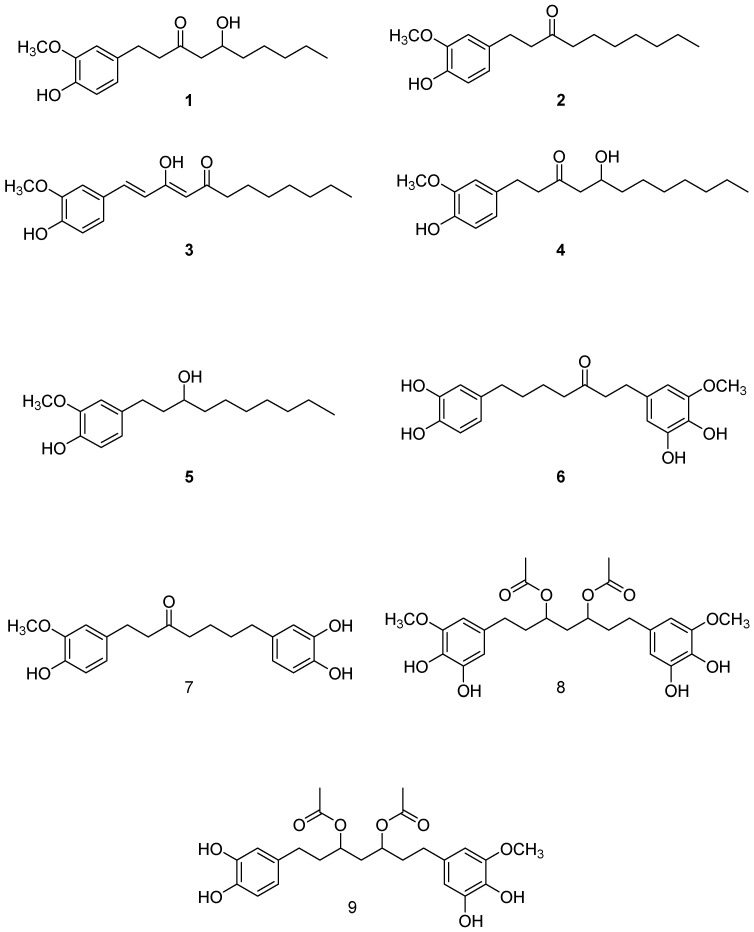
Isolated compounds from *Aframomum meleguta.*

The chloroform soluble fraction of *A. melegueta* seeds was subjected to several chromatographic procedures to obtain nine pure compounds (Figure 1). Five were identified as hydroxyphenyl alkanes: 6-gingerol (**1**) [23], 6-paradol (**2**) [23,24], 8-dehydrogingerdione (**3**) [25], 8-gingerol (**4**) [23], and dihydro-6-paradol (**5**) [26]. This is in addition to four diarylheptanoids identified as dihydrgingerenone A (**6**) [27], dihydrogingerenone C (**7**) [27], 1,7-bis(3,4-dihydroxy-5-methoxyphenyl)heptane-3,5-diyl diacetate (**8**) [24], and 1-(3,4-dihydroxy-5-methoxyphenyl)-7-(3,4-dihydroxyphenyl)heptane-3,5-diyl diacetate (**9**) [24]. The identity of the isolated compounds was confirmed through comparison with the previous literature using their ^1^H- and ^13^ C-NMR data. All the isolated compounds were over 95% pure as indicated from their NMR data, in addition the purity of paradol was over 98% as indicated by HPLC (supplementary data).

### 2.2. Determining Cell Killing Effects and Subcytotoxic Concentration of Compounds Isolated from A. meleguta in MCF-7, MG-63, and SAOS-2 Cell Lines

**Figure 2:**
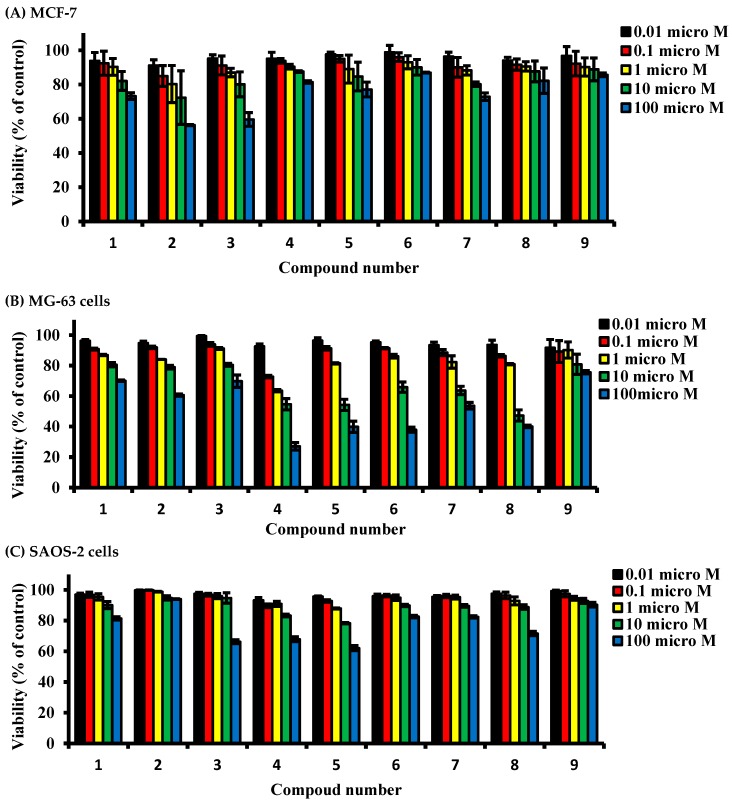
Dose response assessment for compounds isolated from *A. meleguta* against MCF-7 (**A**), MG-63 (**B**) and SAOS-2 cells (**C**). Cells were treated with test compounds for 72 h, and viability was determined using SRB assay. Data are expressed as mean ± SD; *n* = 6.

The cell killing effect of compounds under investigation was tested against three cell lines (MCF-7, MG-63, and SAOS-2 cells) using SRB viability assay to determine sub-cytotoxic concentration for further assessments. Primarily, in the estrogenic MCF-7 cells, all compounds did not alter the cell viability by more than 20% after exposure to concentrations up to 1 µM for 72 h. A higher concentration (10 µM) of paradol (**2**) induced a viability drop to 72.3% of the control untreated cells after exposure for 72 h; other compounds did not induce any viability drop below 80%. Furthermore, exposure of MCF-7 cells to 100 µM of compounds **1**, **2**, **3**, **5**, and **7** for 72 h induced a viability drop to 73.2%, 56.3%, 58.6%, 77.1%, 60.6% and 72.9%, respectively; other compounds did not induce any viability drop below 80% (Figure 2A).

Exposure of MG-63 cells to all compounds under investigation at concentration up to 1 µM for 72 h did not alter the cell viability by more than 20%. A higher concentration (10 µM) of compounds **2**, **4**, **5**, **6**, **7**, and **8** induced a viability drop in MG-63 cells to 79%, 54.6%, 54.2%, 63.7%, 65.8%, 63.7%, and 47.2% of control untreated cells, respectively. Other compounds did not induce any viability drop below 80%. Also, exposure of cells to 100 µM of compounds **3**, **5**, **6**, **7**, and **8** for 72 h induced a cell killing effect by 50% or more compared to control cells. Other compounds induced a viability drop to 60.0–75.7% (Figure 2B).

Interestingly, SAOS-2 cells were the most tolerable cells to the killing effects of the compounds under investigation. Exposure of SAOS-2 to all compounds under investigation (up to 10 µM) for 72 h did not alter the cell viability by more than 17%. Only cells exposed to 100 µM of **3**, **4**, **5**, and **8** suffered from a viability drop to 66.2%, 67.9%, 62%, and 71.6% of control untreated cells, respectively (Figure 2C).

### 2.3. Determining the Proliferative Properties of Compounds Isolated from A. meleguta in MCF-7, MG-63, and SAOS-2 Cell Lines

**Figure 3:**
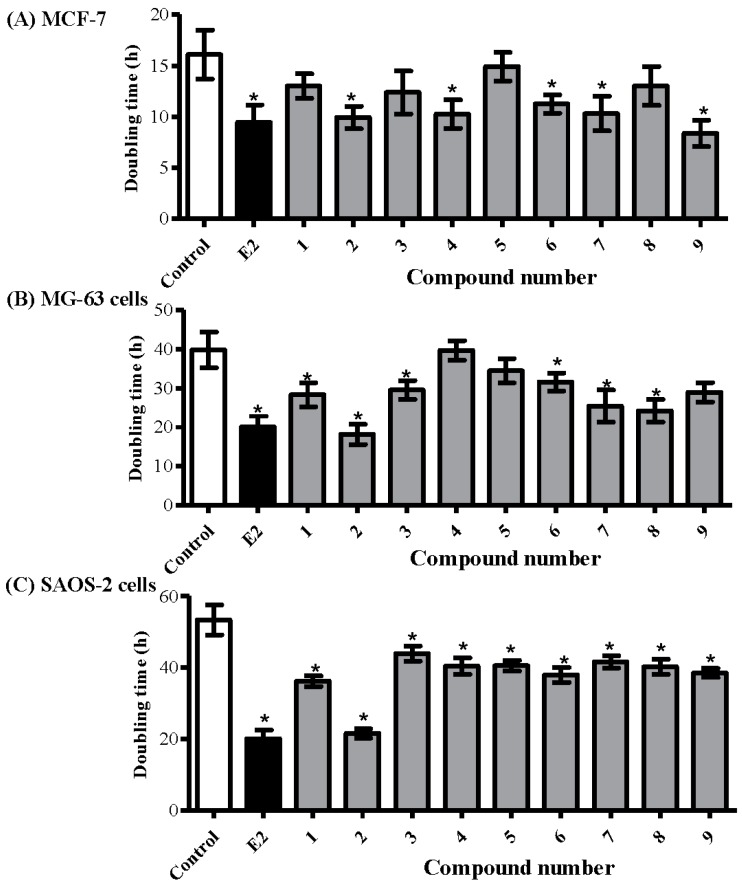
Determining the proliferative effects of compounds isolated from *A. meleguta* using MCF-7 (**A**); MG-63 (**B**); and SAOS-2 (**C**) cell lines. Cells were treated with test compounds (1 µM) for up to 96 h, and viability was determined using SRB assay. Doubling times were calculated and compared to control untreated cells and E_2_ (0.1 µM) treated cells (positive control). Data are expressed as mean ± SD; *n* = 6. * Significantly different from untreated cells; *p* < 0.05.

The proliferative effect of compounds under investigation was tested in the aforementioned cell lines (MCF-7, MG-63, and SAOS-2) using the predetermined sub-cytotoxic dose (1 µM) and compared to estradiol (E_2_) 0.1 µM as a positive control. The doubling time of MCF-7 cells was calculated with/without the presence of the compounds under investigation. Compounds **2**, **4**, **6**, **7**, and **9** significantly decreased the doubling time of MCF-7 from 16.1 ± 2.1 h to 9.9 ± 1.1 h, 10.3 ± 1.4 h, 11.3 ± 0.9 h, 10.3 ± 1.7 h, and 8.4 ± 1.4 h, respectively. MCF-7 treatment with E_2_ decreased its doubling time to 9.5 ± 1.7 h (Figure 3A).

In terms of bone osteosarcoma-derived cell lines, compounds **1**, **2**, **3**, **6**, **7**, and **8** decreased the doubling time of MG-63 cells from 39.9 ± 4.7 h to 28.3 ± 3.1 h, 18.3 ± 2.6 h, 29.5 ± 2.4 h, 31.6 ± 2.3 h, 25.4 ± 4.1 h, and 24.2 ± 2.9 h, respectively. Yet, E_2_ decreased the doubling time of MG-63 cells to 20.1 ± 2.1 h (Figure 3B). Interestingly, all compounds under investigation, including **6**, significantly decreased the doubling time of SAOS-2 cells. Among them, **2** showed the most prominent decrease in doubling time from 53.3 ± 4.2 h to 21.5 ± 1.3 h, respectively, compared to 20.1 ± 2.4 h by E_2_ (Figure 3C).

Among the compounds under investigation, paradol (**2**) showed promising proliferative and potential antiosteoporotic properties. From another aspect, and due to its minimal change of viability in response to treatment with all compounds under investigation, SAOS-2 cells were used for further subsequent assessments.

### 2.4. Determining the Effect of Paradol on Cell Cycle Distribution

**Figure 4:**
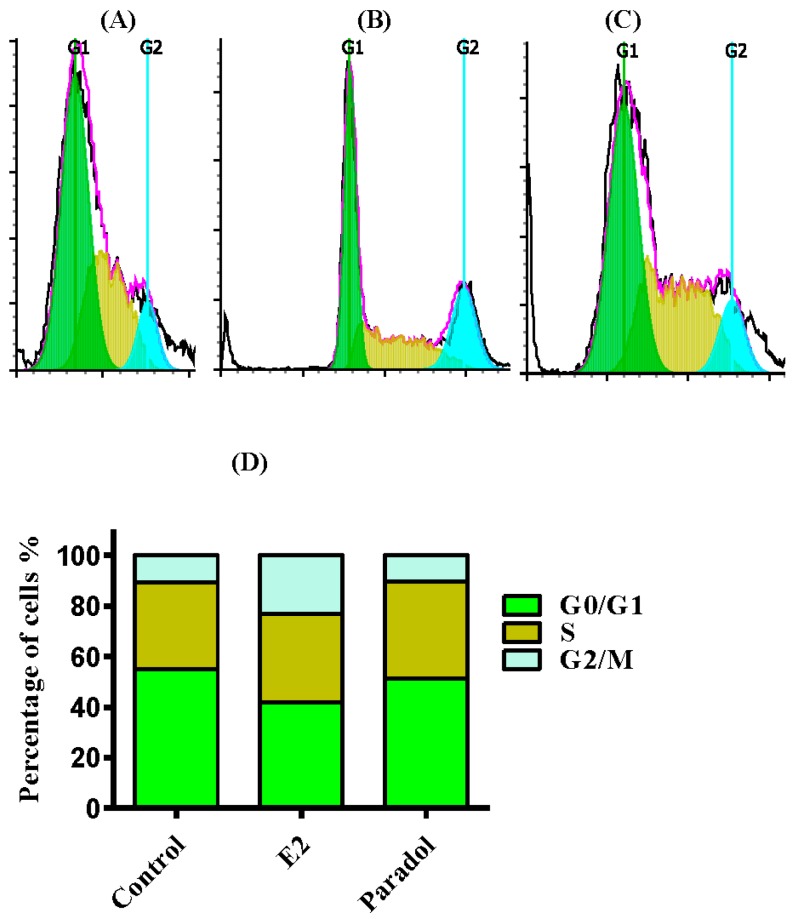

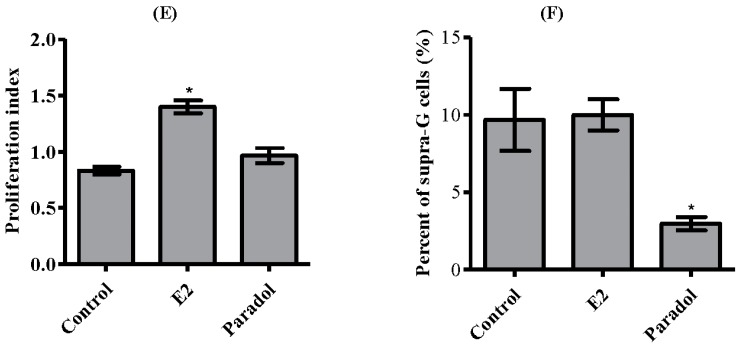
Effect of paradol on the cell cycle distribution of SAOS-2 cells. Cells were exposed to paradol (1 µM) for 24 h (**B**) and compared to control untreated cells (**A**) and E_2_ (0.1 µM) treated cells (**C**); Cell cycle distribution was determined using DNA flow cytometric analysis, and different cell phases were plotted (**D**) as the percentage of total events. The proliferation index was calculated and plotted (**E**); Supra-G_2_/M cell population was plotted as the percent of total events (**F**). Data are presented as mean ± SD; *n* = 3. * Significantly different from control untreated cells; *p* < 0.05.

To test whether paradol’s effect on cell proliferation is accompanied by a disturbance in cell cycle phase distribution and cell cycle progression, DNA content flow-cytometry was undertaken after treating SAOS-2 cells with paradol and comparing to E_2_ (positive control) for 24 h. Paradol (1 µM) did not exert any significant change in the G_0_/G_1_ or G_2_/M phases of SAOS-2 cells after treatment for 24 h; however, it marginally increased the percentage of cells in the S-phase from 34.3 ± 0.4% to 38.4 ± 1.8% (Figure 4A,B,D). On the other hand, E_2_ (0.1 µM) significantly increased cells in the G_2_/M-phase (from 10.9 ± 1.5% to 23.1 ± 1.7%) with reciprocal decrease of the cell population in G_0_/G_1_ (from 54.8 ± 1.9% to 41.9 ± 2.4%). No significant change of cells in the S-phase was detected in SAOS-2 cells after treatment with E_2_ (Figure 4A,C,D). Further assessment of the proliferation cell fraction balance, paradol did not induce any significant change in the proliferation index of SAOS-2 cells; however, E_2_ significantly alters the proliferation index of SAOS-2 cells (Figure 4E). These data, along with the previous finding of decreasing the doubling time of SAOS-2 due to treatment with paradol, indicate a punctuated proliferative effect of paradol (1 µM) without disturbing cell cycle phases in contrast to the classic estrogenic agent, E_2_. To further confirm the controlled effect of paradol on cell proliferation, we assessed the cell population accumulated in supra-G_2_ compartment (multiploidy phase), which is a direct indication for uncontrolled cell cycle progression. Interestingly, paradol significantly decreased the cell population in the Supra-G_2_ phase in contrast to E_2_ (Figure 4F).

### 2.5. Assessment of Osteogenic Gene Expression

**Figure 5:**
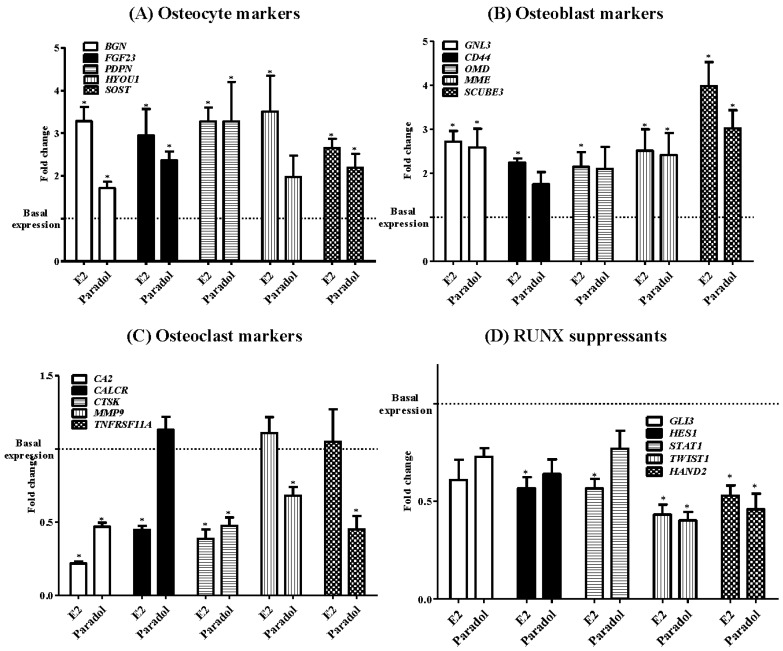
Effect of paradol on mRNA expression of some ossification-related genes in SAOS-2 cell line. Cells were incubated with paradol (1 uM) or E_2_ (0.1 uM) for 48 h. Total RNA was extracted and subjected to RT-PCR. Data were normalized to β-actin; fold changes were calculated and expressed as mean ± SD; *n* = 3. * Significantly different from control untreated cells; *p* < 0.05.

The influence of paradol on the expression of different osteogenic markers was assessed quantitatively using a functional RT-PCR gene array system with four different osteogenic gene sub-families (osteocyte activity markers, osteoblast activity markers, osteoclast activity markers, and RUNX suppressor genes). With respect to osteocyte activity markers (*BGN*, *FGF23*, *PDPN*, *HYOU1*, and *SOST*), paradol increased the expression levels of *BGN*, *FGF23*, *PDPN*, and *SOST* mRNA by 1.7 ± 0.2, 2.4 ± 0.3, 3.3 ± 1.3, and 2.2 ± 0.5 folds, respectively. However, E_2_ increased the expression of all five osteocyte activity gene markers by 2.6 to 3.5 folds (Figure 5A). Similarly, paradol increased the expression level of three osteoblast activity marker mRNA, *GNL3*, *MME*, and *SCUBE3*, by 2.6 ± 0.6, 2.4 ± 0.7, and 3.0 ± 0.6 folds, respectively. The expression of *CD44* and *OMD* did not show any significant change. However, E_2_ increased the expression of all five osteoblast activity gene markers by 2.1 to 3.9 folds (Figure 5B). On the other hand, paradol decreased the expression levels of four osteoclast gene markers, *CA2*, *CTSK*, *MMP9*, and *TNFRF11A*, to 0.5 ± 0.04, 0.5 ± 0.08, 0.7 ± 0.08, and 0.5 ± 0.01 folds of the control level, respectively. No significant change in the expression of *CALCR* gene was detected. E_2_ downregulated the expression of *CA2*, *CALCR*, and *CTSK* to be 0.2 to 0.5 folds of the control level (Figure 5C). With respect to the *RUNX* suppressor gene family (*GLB*, *HES1*, *STAT1, TWIST1*, and *HAND2*), paradol only downregulated the expression of *TWIST1* and *HAND2* mRNA to be 0.4 ± 0.07 and 0.50 ± 0.10 folds of control cells, respectively. However, E_2_ suppressed the expression level of *HES1*, *STAT1*, *TWIST1*, and *HAND2* to be 0.4 to 0.6 folds of the control level (Figure 5C).

### 2.6. Assessment of Antiosteoporotic Biochemical Markers

**Figure 6:**
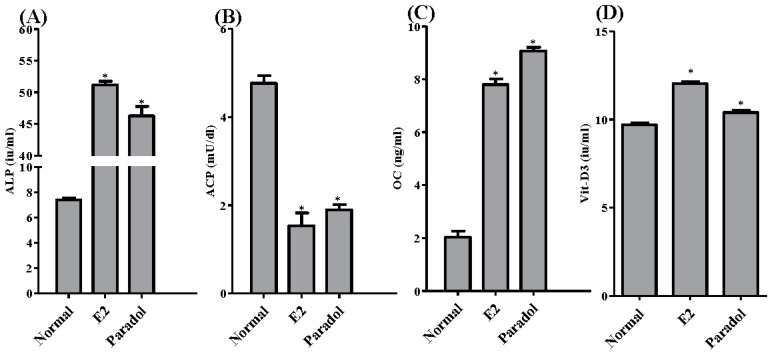
Biochemical assessment for antiosteoporotic effect of paradol in vitro. SAOS-2 cells were treated with paradol (1 uM) and E_2_ (0.1 uM) for 48 h and compared to control untreated cells. Biochemical assessment of osteoporosis was evaluated by measuring alkaline phosphatase (**A**); acid phosphatase (**B**); osteocalcin (**C**); and vitamin-D3 (**D**). Data are presented as mean ± SD; *n* = 3. * Significantly different from control untreated cells; *p* < 0.05.

The activity/concentrations of four important ossification-related markers was/were assessed. This was performed in the media of SAOS-2 cells after treatment for 48 h with paradol (1 µM) compared to E_2_ (0.1 µM) as a positive control. Paradol significantly increased the activity of the ALP enzyme (ossification marker) from 7.4 ± 0.3 i.u./mL to 46.3 ± 2.6 i.u./mL compared to 51.2 ± 1.0 i.u./mL for treatment with E_2_ (Figure 6A). Conversely, paradol significantly decreased the activity of ACP enzyme (bone resorption marker) from 4.8 ± 0.3 i.u./mL to 1.9 ± 0.2 i.u./mL compared to 1.5 ± 0.5 i.u./mL for treatment with E_2_ (Figure 6B). Similar to ALP, paradol significantly increased the expression of a key ossification hormone—osteocalcin—from 9.1 ± 0.3 ng/mL to 2.0 ± 0.4 ng/mL compared to 7.8 ± 0.4 ng/mL for treatment with E_2_ (Figure 6C). In alignment with these ossification markers, paradol also increased the concentration of 1,25-dihydroxy calciferol (Active Vit-D3) from 9.8 ± 0.1 i.u./mL to 10.4 ± 0.2 i.u./mL compared to 12.0 ± 0.2 i.u./mL for treatment with E_2_ (Figure 6D).

## 3. Discussion

Osteoporosis is a serious health problem that also poses a heavy social and economic burden [28]. Current antiosteoporotic agents are not devoid of adverse effects, which range from gastrointestinal irritation to carcinogenesis [7,29]. Due to their known safety profile and wide acceptance, phytochemicals are being recommended for prevention of osteoporosis. Further, they are attractive leads for developing a synthetic series of novel agents against bone loss [30]. El-Halawany et al. [18] reported that *A. melegueta* extract possesses potent estrogen-modulating and thus potential antiosteoporotic activity. Therefore, the current study aimed to isolate and identify phytochemicals from *A. melegueta* with superior proliferative and ossification characteristics in bone cells.

The isolated compounds from *A. melegueta* were tested herein to show promising proliferative effects in three different estrogen-dependent cell lines (MCF-7, MG-63, and SAOS-2). Previous work showed the ability of estrogen and some of its metabolites to induce proliferation; however, the major limitation was their mutagenic ability [31,32]. Compounds of these two chemical families (hydroxyphenylalkanes and diarylheptanoids) such as gingerol, shogoal, and paradol are well known due to their anticarcinogenic properties [33,34]. It was very interesting to see the proliferative effect of paradol against estrogen-dependent cell lines without disturbing the balance between the different cell cycle phases. Paradol did not affect the proliferation index and, in contrast, decreased the percent of cells in the multiploidy phase. Yet, accumulating cells in the multiploidy phase is indicative of improper and pro-carcinogenic features [35].

It is worth mentioning that ossification, more than proliferation, is clinically impactful for treating osteoporosis [36,37,38]. Herein, we assessed the impact of paradol on mRNA expression of different ossification-related gene clusters. The osteocyte activity-related genes BGN, FGF23, PDPN, and SOST were significantly increased by exposure to paradol. Osteocytes constitute 90% to 95% of the all bone cells. They regulate activities of the osteoblasts and osteoclasts [39]. The integrated effects of the osteocytic markers highlight the regulatory role of osteocytes in mineral metabolism and phosphate homeostasis. Osteocytes cloned with BGN significantly accelerated osteoblast differentiation. Furthermore, transplanting these clones into immunodeficient mice resulted in larger areas of lamellar bone-like matrices [40]. FGF23 protein functions to reduce phosphate levels. FGF23 expression in osteocytes is upregulated in several diseases, including osteomalacia [41]. PDPN is another marker of osteocyte activity and highly expressed in dividing osteocytes [42]. The paracrine functions of osteocytes include secretion of SOST, which negatively influences bone formation [43]. The exact role of HYOU1 in the biology of osteocytes is yet to be revealed. The observed increased expression of SOST and HYOU1 cannot be explained. The potential beneficial effects of paradol were confirmed by the observed increase in mRNA expression of the osteoblast activity markers GNL3, CD44, OMD, MME, and SCUBE3 as well as the inhibited expression of the osteoclast activity markers CA2, CALCR CTSK, MMP9, and TNFRF11A. RUNX2 has been shown to play a crucial role in osteoblast differentiation and maturation by regulating RANKL [44]. The potential osteogenic effects of paradol were further highlighted by the decreased expression of the RUNX suppressors GLB, HES1, STAT1, TWIST1, and HAND2. Besides influencing ossification gene expression, paradol increased the concentration/activity of ALP, OC, and the active form of Vit-D with a reciprocal decrease in ACP. Ultimately, paradol resulted in clear biochemical evidence for shifting the ossification balance toward calcium deposition in bones rather than bone resorption. These biochemical markers are well documented and used as markers for antiosteoporotic activity [45]. Conclusively, the phytochemical analysis of *A. melegueta* extract resulted in the separation and identification of nine compounds belonging to hydroxyphenylalkanes and diarylheptanoids groups with potential antiosteoporotic properties. Among them, paradol exhibited the most potent proliferation and ossification characteristics in bone cells.

## 4. Materials and Methods

### 4.1. General

Nuclear magnetic resonance (NMR, ^1^H, 400 MHz; ^13^C, 100 MHz) spectra were recorded on a JHA-LAA 400 WB-FT spectrometer (Jeol Co., Tokyo, Japan). Thin layer chromatography (TLC) precoated silica gel 60 F_254_, and reversed phase (RP)-18 F_254_S 0.25 mm, plates were purchased from Merck Co., Dermstadt, Germany. Column chromatography (CC) was carried out on BW-820MH silica gel, Wakosil C-300 (40–64 μm) (Wako, Osaka, Japan). Medium pressure liquid chromatography (MPLC), LiChroprep RP-18 ready-made columns (size A and B) were purchased from Merck Co. (Darmstadt, Germany)

### 4.2. Plant Material

Seeds of *A. melegueta* were identified by Dr. Sherif El-Khanagry, Agriculture Museum, Dokki, Giza. A specimen was kept in the herbarium of the Department of Pharmacognosy, Faculty of Pharmacy, King Abdulaziz University.

### 4.3. Extraction and Isolation

Powdered seeds of *A. melegueta* (2.0 kg) were extracted with methanol using Ultratorrux at room temperature, and the pooled methanol extracts were evaporated using a rotatory evaporator to yield a semisolid brownish residue (100 g). The total methanol extract was suspended in water (500 mL) and partitioned with CHCl_3_ (1 L × 3) to yield, after evaporation, a CHCl_3_-soluble fraction (55 g). The remaining aqueous layer was freeze-dried and kept for further investigation. The CHCl_3_ fraction was chromatographed on a silica gel column (70 × 8 cm) using *n*-hexane-EtOAc gradiently from 5 until 80% *v/v*. The fractions obtained from the column were pooled based on TLC investigations into 10 sub-fractions (F1–F10). F1 (11 g) was chromatographed on a silica gel column (40 × 4 cm) eluted with hexane–EtOAc (9.5:0.5 *v/v*) to obtain pure compound **2** (9 g). Compound **1** was isolated in a pure form (4 g) after applying F3 (6 g) to a silica gel column using hexane–EtOAc (9:1 *v/v*). The remaining of F3 was pooled into eight sub-fractions (3-1~3-8). Fraction 3-5 (1 g) was purified on MPLC-RP18 column size B using MeOH:H_2_O (8:2 *v/v*) as a mobile phase to yield compound **3** (15 mg). Fractions 3-7 and 3-8 were treated in a similar way to fraction 3-5 using the mobile phase MeOH:H_2_O (6:4 *v/v*) to attain compounds **4** and **5** (5 and 25 mg), respectively. Sub-fraction 9 (10 g) was fractionated on a silica gel column using hexane–EtOAC (9:1~5:5 *v/v*) to obtain compound **9** (200 mg) in addition to five sub-fractions. Sub-fraction 9-5 (500 mg) was placed on MPLC-RP 18 column size A and eluted with MeOH–H_2_O (6:4 *v/v*) to yield compounds **6** (5 mg) and **7** (7 mg). Fraction 10 (3.5 g) was chromatographed on a silica gel column using hexane–EtOAc (6:4 *v/v*) followed by purification on a MPLC-RP 18 column using MeOH–H_2_O (1:1 *v/v*) to afford **8** (10 mg).

### 4.4. Chemicals and Media

Sulfarhodamine B (SRB), propidium iodide (PI), RNAase-A enzyme, and 17β-hydroxyesradiol were purchased from Sigma–Aldrich Chemical Company (St. Louis, MO, USA). DMEM media, McCoy’s-5A media, MEM media, fetal bovine serum, and other cell culture materials were purchased from Gibco™, Thermo Fisher scientific (Waltham, MA, USA). All other chemicals were of the highest available analytical grade.

### 4.5. Cell Culture 

Human estrogen-dependent breast adenocarcinoma cells (MCF-7 cell line) and human osteosarcoma cell lines (MG-63 and SAOS-2) were obtained from the VACSERA (Giza, Egypt). Cells were maintained in DMEM, McCoy’s-5A and MEM media, respectively. Media were supplemented with 100 µg/mL streptomycin, 100 units/mL penicillin, and 10% heat-inactivated fetal bovine serum and used to feed cells in a humidified, 5% (*v/v*) CO_2_ atmosphere at 37 °C.

### 4.6. Cytotoxicity Assessment 

Cytotoxicity of compounds isolated from *A. meleguta* was performed on MCF-7, MG-63, and SAOS-2 cells by SRB-assay, as previously described [46]. Briefly, exponentially growing cells were collected using 0.25% Trypsin-EDTA and plated in 96-well plates at 1000–2000 cells/well. Cells were exposed to test compounds for 72 h and subsequently fixed with TCA (10%) for 1 h at 4 °C. After washing thrice, cells were exposed to a 0.4% SRB solution for 10 min in the dark and subsequently washed with 1% glacial acetic acid. After drying overnight, Tris-HCl was used to dissolve the SRB-stained cells, and color intensity was measured at 540 nm.

### 4.7. Proliferation Assay (Doubling Time Determination)

The proliferative effect of compounds isolated from *A. meleguta* was tested in MCF-7, MG-63, and SAOS-2 cells by SRB assay. Briefly, exponentially growing cells were exposed to sub-cytotoxic concentration (1 µM) of the isolated compounds in phenol red free media for 24, 48, 72, and 96 h. Cells were subsequently stained with SRB solution for quantification, and the time required for the cell count to double (doubling time) was calculated from the best fit linear regression curve [31].

### 4.8. Analysis of Cell Cycle Distribution

To assess the effect of the selected active compound (paradol) on cell cycle distribution, SAOS-2 cells were treated with 1 µM paradol for 24 h and 48 h and compared to estradiol (0.1 µM). After treatment, cells were collected by trypsinization and washed twice with ice-cold PBS and re-suspended in 0.5 mL of PBS. Two milliliters of 70% ice-cold ethanol were added gently while vortexing. Cells were kept in an ethanol solution at 4 °C for 1 h for fixation. Upon analysis, fixed cells were washed and re-suspended in 1 mL of PBS containing 50 μg/mL RNAase-A and 10 μg/mL propidium iodide (PI). After a 20-min incubation in a dark place at room temperature, cells were analyzed for DNA contents. Cells were injected through ACEA Novocyte™ flow-cytometer (ACEA Biosciences Inc., San Diego, CA, USA) and analyzed for PI fluorescent signals using an FL2 signal detector (λex/em 535/617 nm). For each sample, 12,000 events were acquired, and cell cycle phases were quantified by using ACEA NovoExpress™ software (ACEA Biosciences Inc., San Diego, CA, USA, version 1.1.0) after defining the cell fragment-free fluorescent gate. Ungated events were used to determine cells in the supra-G_2_/M phase. The proliferation index was calculated by dividing the total cells in S- and G_2_/M-phases by cells in G_0_/G_1_ phases.

### 4.9. Osteogenic Gene Array Analysis

To assess the gene expression of several osteogenic/osteolytic related genes, cells (1 × 10^6^) were treated with paradol (1 µM) or estradiol (0.1 µM) as a positive control for 48 h. The total RNA was extracted from cells using RNeasy Mini Kit^®^ (Qiagen Inc., Valencia, CA, USA), and reverse transcription was undertaken to construct the cDNA library from different treatments using the High-Capacity cDNA Reverse Transcription Kit (Applied Biosystems, Foster City, CA, USA). The archived cDNA libraries were then subjected to quantitative real-time PCR reactions using GeneQuery™ Human Osteogenic Differentiation qPCR Array (Science Cell Research Laboratories Inc., Carlsbad, CA, USA) according to manufacturer protocol [47]; the β-actin gene was used as a housekeeping background gene. The normalized fold change of gene expression for all genes of interest after paradol and estradiol treatment was calculated using the formula: 2^−ΔΔ*C*q^.

### 4.10. Assessment of Biochemical Osteoporosis Marker

To assess the influence of paradol on the biochemical ossification markers secreted from SAOS-2, 1 × 10^6^ cells were treated with paradol (1 µM) or estradiol (0.1 µM) as a positive control. After 48 h, media were collected and assayed for the level of alkaline phosphatase (ALP), acid phophatase (ACP), osteocalcin (OC), and the active form of Vit-D. ALP and tartrate-resistant ACP were determined by direct HTS-ready colorimetric assay (Abcam, Cambridge, UK) [48]. Osteocalcin was determined using Uscan^®^ immunoassay ELISA Kit (Life Science Inc., Wuhan, China) according to the manufacturer’s instructions [49]. An active form of Vit-D was determined using Human Total 25-OH Vitamin D IVD ELISA Kit (R&D Systems, Inc., Minneapolis, MN, USA).

### 4.11. Statistical Analysis

Data are presented as mean ± SEM. For multiple comparisons, two-way analysis of variance (ANOVA) followed by Bonferroni post hoc was used for testing the significance by SPSS^®^ for Windows, version 17.0.0. *p* < 0.05 was taken as a cutoff value for significance.

## Supplementary Materials

Supplementary materials are available online.
