# Supplementary material for: Phenolics Isolated from Aframomum meleguta Enhance Proliferation and Ossification Markers in Bone Cells
Source: Molecules. 2017 Sep 4;22(9):1467. doi: 10.3390/molecules22091467 (PMC6151453; doi:10.3390/molecules22091467)
Supplement: Supplementary file 1 [file molecules-22-01467-s001.pdf]

Supplementary data

Phenolics isolated from *Aframomum meleguta* enhance proliferation and ossification markers in bone cells

Ashraf B. Abdel-Naim [1,2], Abdullah A. Alghamdi [3], Mardi M. Algandaby [1,4], Fahad A. Al-Abbasi [1,3], Ahmed M. Al-Abd [2,5], Hossam M. Abdallah [6,7], Ali M. El-Halawany [7], Masao Hattori [8]

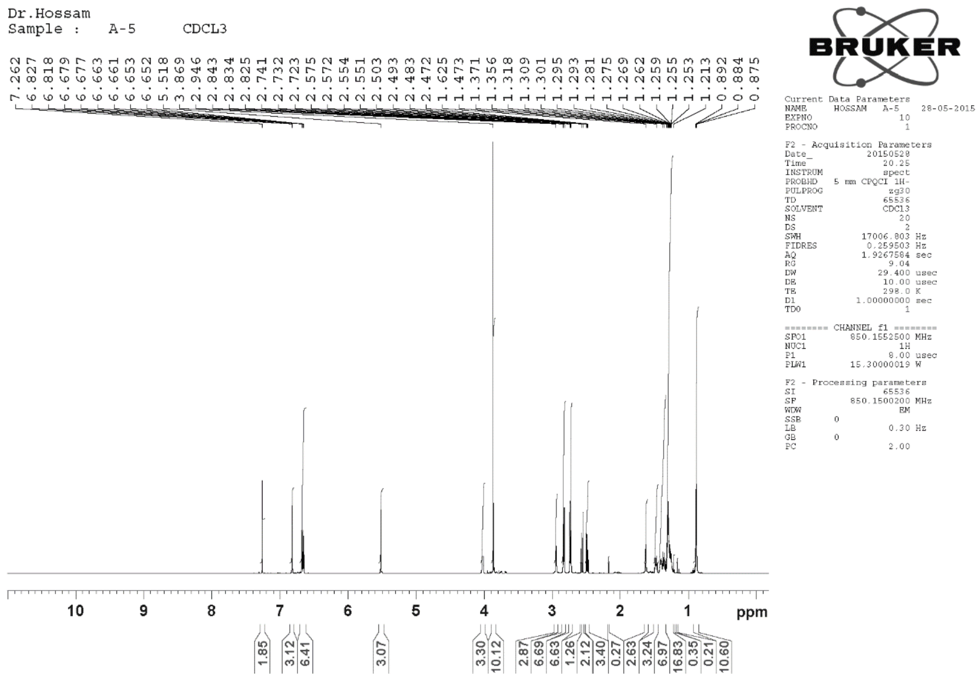

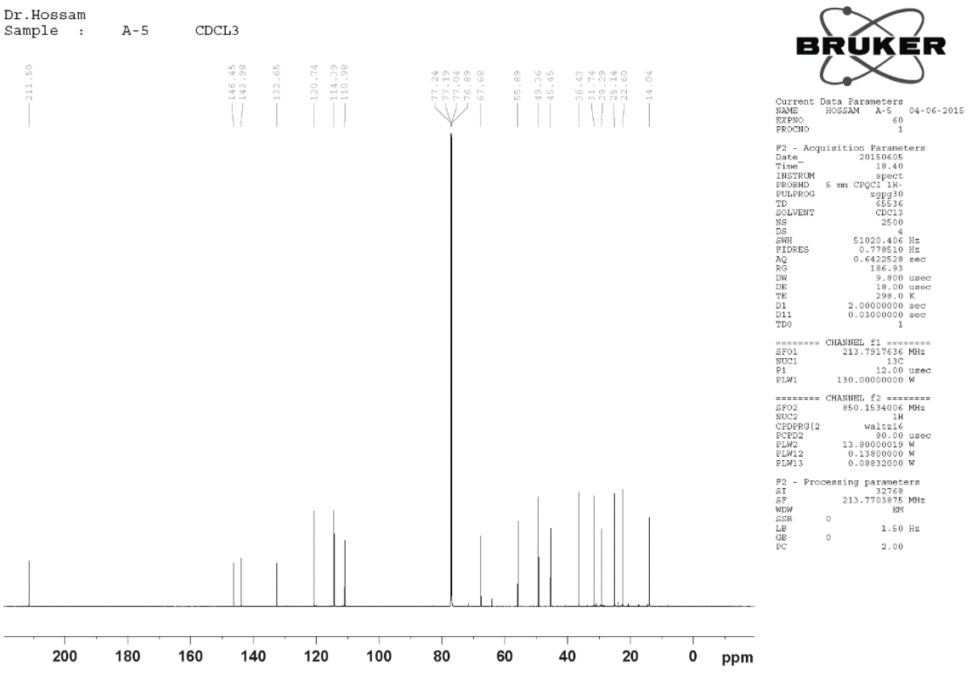

Figure S1. ^{1}H and ^{13} C NMR charts of **6-gingerol**

(Samples were recorded on DRX-850 spectrometer (Bruker BioSpin, Billerica,MA, USA).

Dr.Hossam
Sample : PARADOL CDCL3

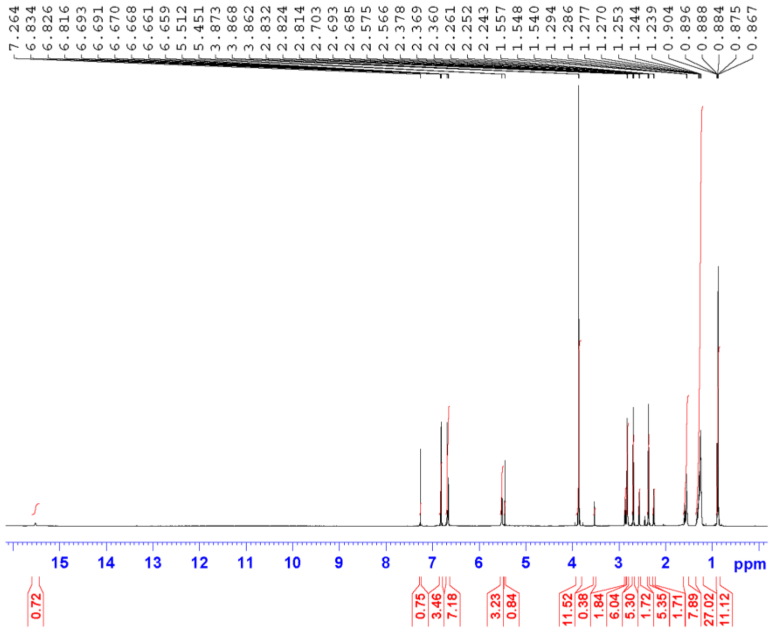


```
Current Data Parameters
NAME      HOSSAM    PARAD     21-01-2015
EXPNO               10
PROCNO              1

F2 - Acquisition Parameters
Date_          20150121
Time              13.40
INSTRUM           spect
PROBHD   5 mm CPQCI 1H-
PULPROG            zg30
TD                65536
SOLVENT           CDCl3
NS                   32
DS                    2
SWH           17006.803 Hz
FIDRES         0.259503 Hz
AQ            1.9267584 sec
RG                 7.85
DW               29.400 usec
DE                10.00 usec
TE                298.0 K
D1           1.00000000 sec
TD0                   1

======== CHANNEL f1 ========
SFO1        850.1552500 MHz
NUC1                 1H
P1                 8.00 usec
PLW1        15.30000019 W

F2 - Processing parameters
SI                65536
SF          850.1500185 MHz
WDW                  EM
SSB      0
LB                 0.30 Hz
GB       0
PC                 2.00
```

Dr.Hossam
Sample : PARADOL CDCL3

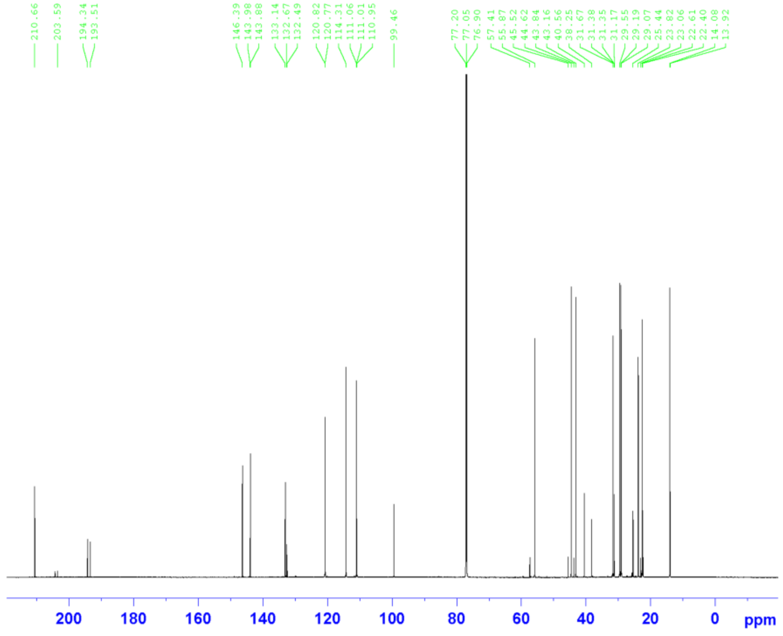


```
Current Data Parameters
NAME      HOSSAM    PARADOL   27-01-2015
EXPNO               40
PROCNO              1

F2 - Acquisition Parameters
Date_          20150127
Time              18.52
INSTRUM           spect
PROBHD   5 mm CPQCI 1H-
PULPROG          zgpg30
TD                65536
SOLVENT           CDCl3
NS                 4096
DS                    4
SWH           51020.406 Hz
FIDRES         0.778510 Hz
AQ            0.6422528 sec
RG               186.93
DW                9.800 usec
DE                18.00 usec
TE                298.0 K
D1           2.00000000 sec
D11          0.03000000 sec
TD0                   1

-------- CHANNEL f1 --------
SFO1        213.7917636 MHz
NUC1                13C
P1                12.00 usec
PLW1       130.00000000 W

-------- CHANNEL f2 --------
SFO2        850.1534006 MHz
NUC2                 1H
CPDPRG[2        waltz16
PCPD2             80.00 usec
PLW2        13.80000019 W
PLW12        0.13800000 W
PLW13        0.08832000 W

F2 - Processing parameters
SI                32768
SF          213.7703875 MHz
WDW                  EM
SSB      0
LB                 1.50 Hz
GB       0
PC                 2.00
```

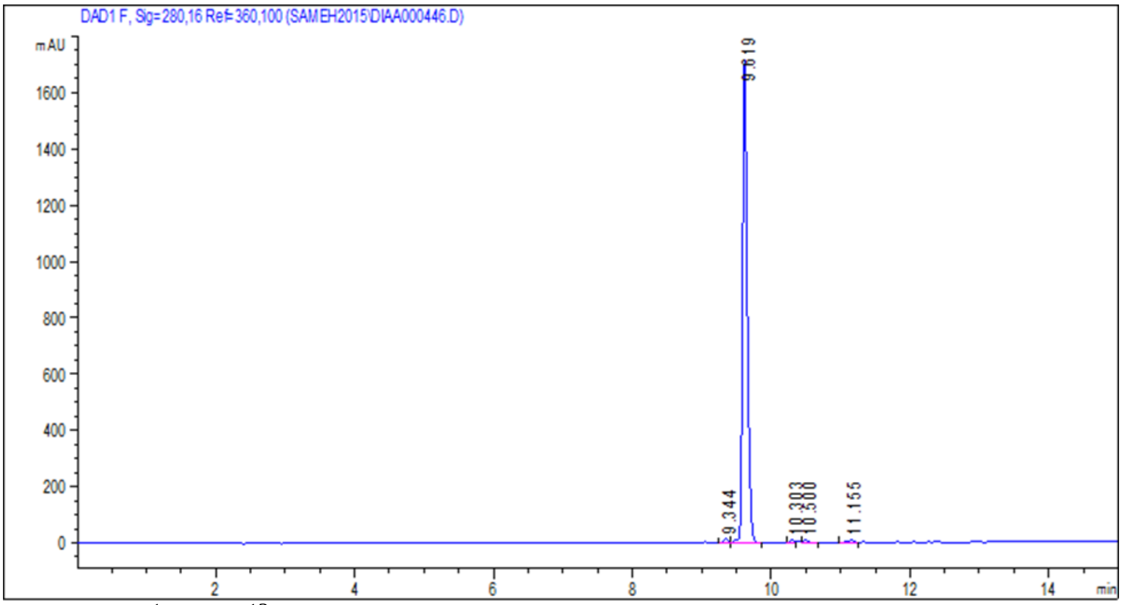

Figure S2. ^{1}H and [13] C NMR charts and HPLC chromatogram of **6-paradol**
(Samples were recorded on DRX-850 spectrometer (Bruker BioSpin, Billerica,MA, USA).

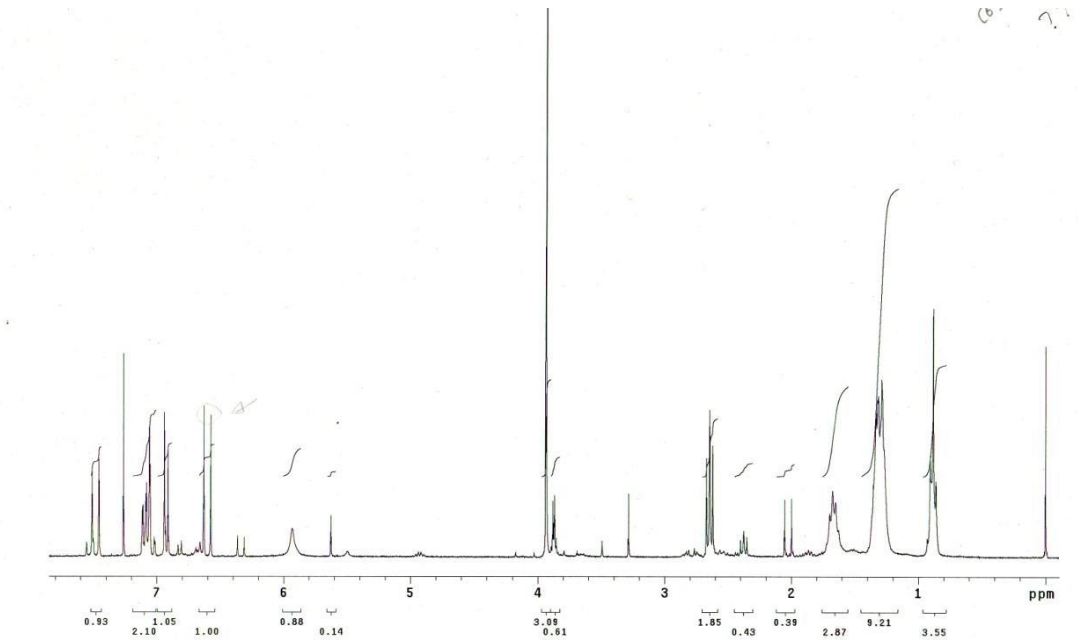
7
6
5
4
3
2
1
ppm
0.93
2.10
1.05
1.00
0.88
0.14
3.09
0.61
1.85
0.43
0.39
2.87
9.21
3.55

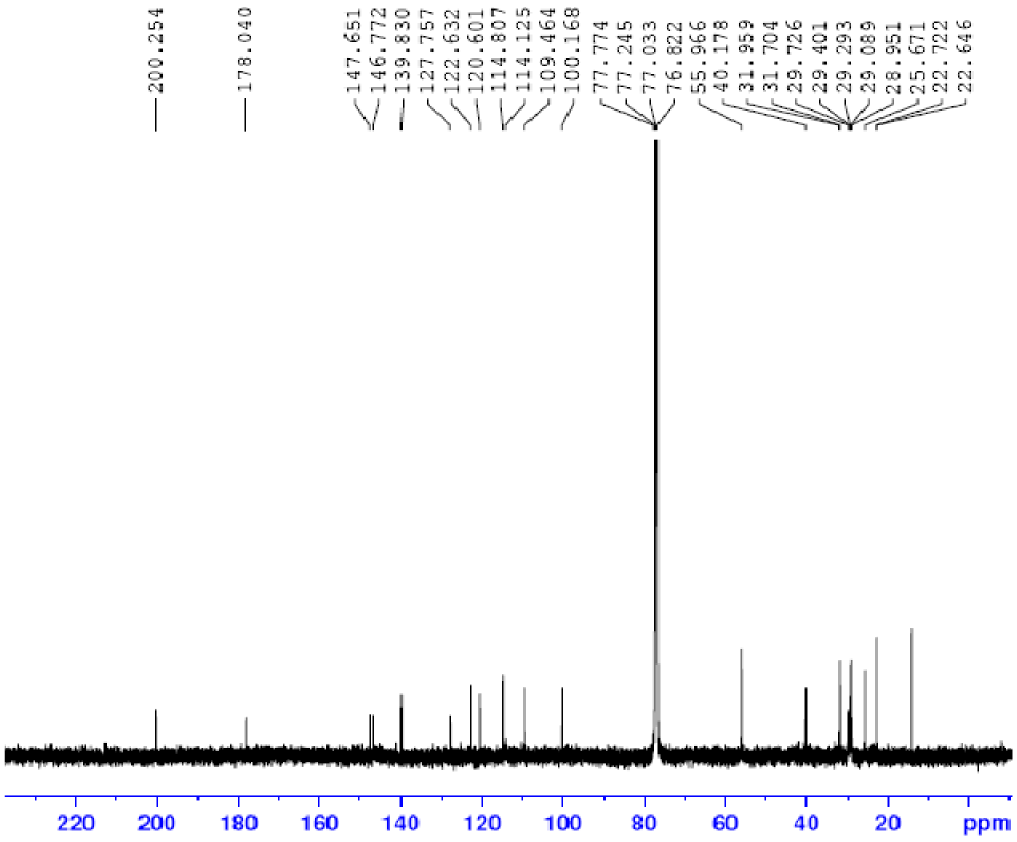
200.254
178.040
147.651
146.772
139.830
127.757
122.632
120.601
114.807
114.125
109.464
100.168
77.774
77.245
77.033
76.822
55.966
40.178
31.959
31.704
29.726
29.401
29.293
29.089
28.951
25.671
22.722
22.646
220
200
180
160
140
120
100
80
60
40
20
ppm

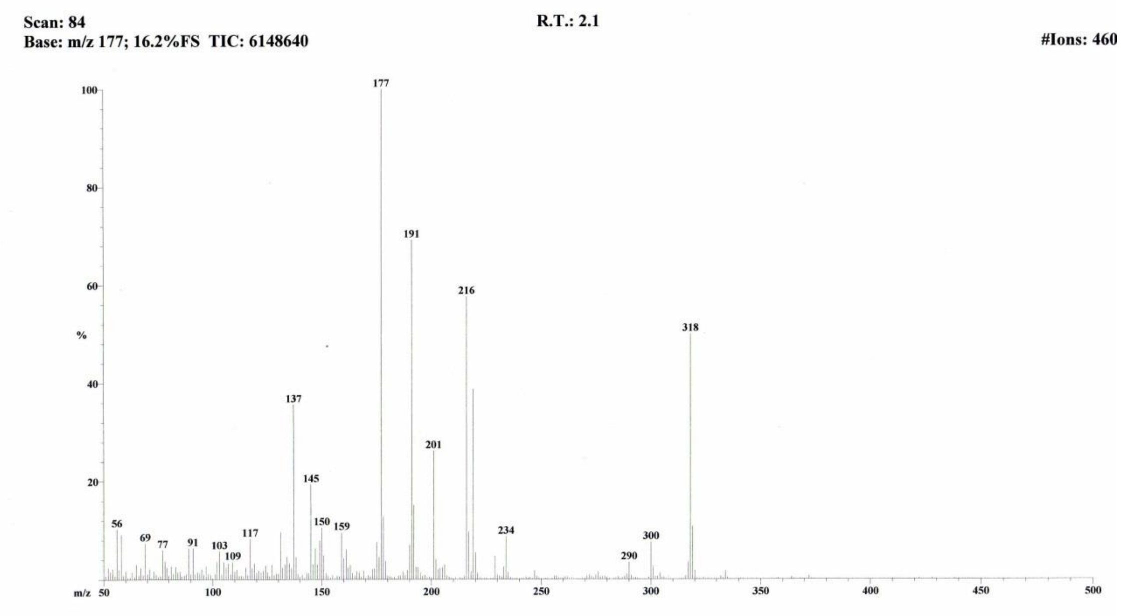

Figure S3. ^{1}H and [13] C NMR and Ms Charts of 8-**dehydrogingerdione (3)**

3-7-3

[8] gingerol

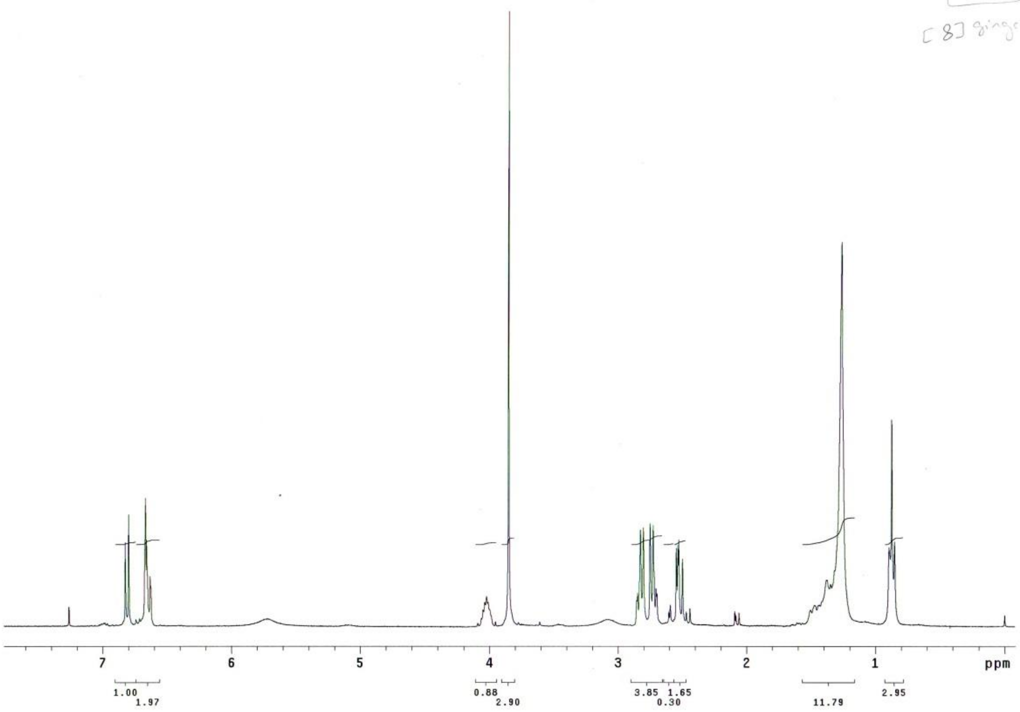

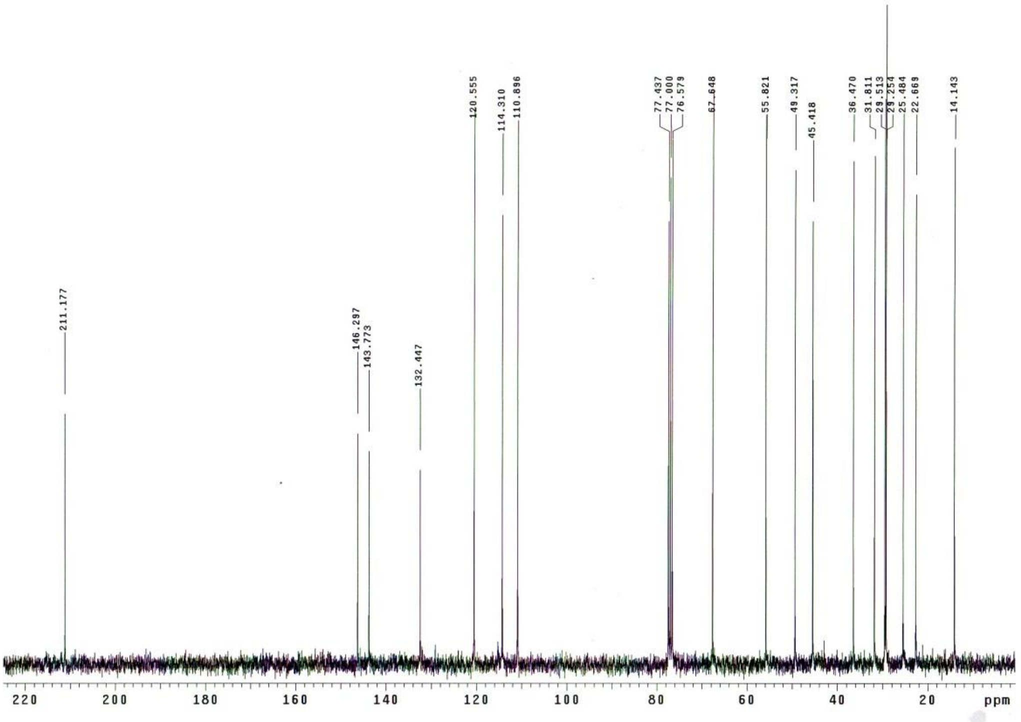

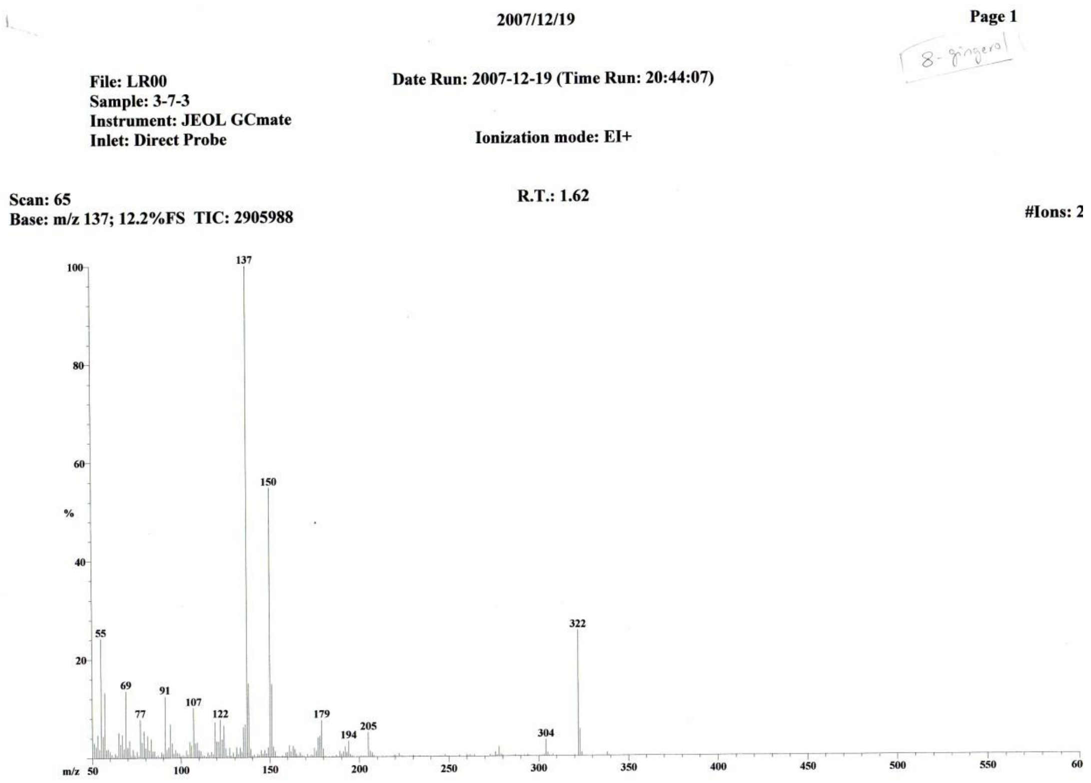

Figure S4. ^{1}H and ^{13} C NMR and MS charts of **8-gingerol (4)**

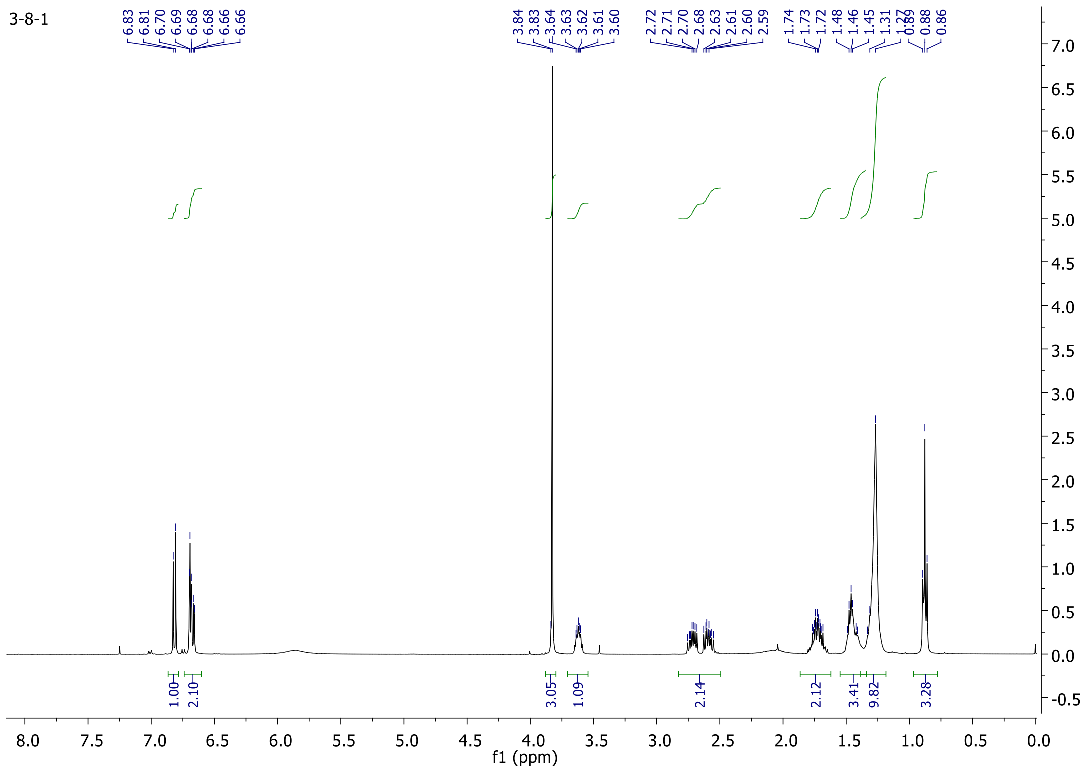

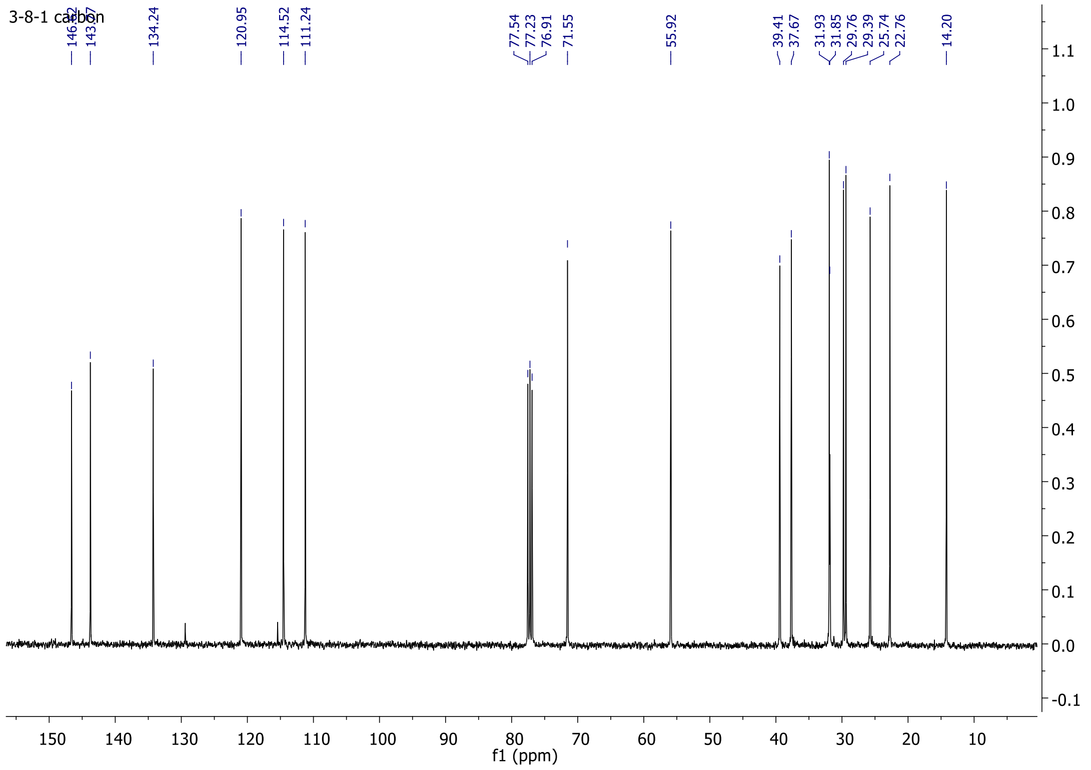

Figure S5. ^{1}H and ^{13}C NMR charts of **dihydro-6-paradol (5)**

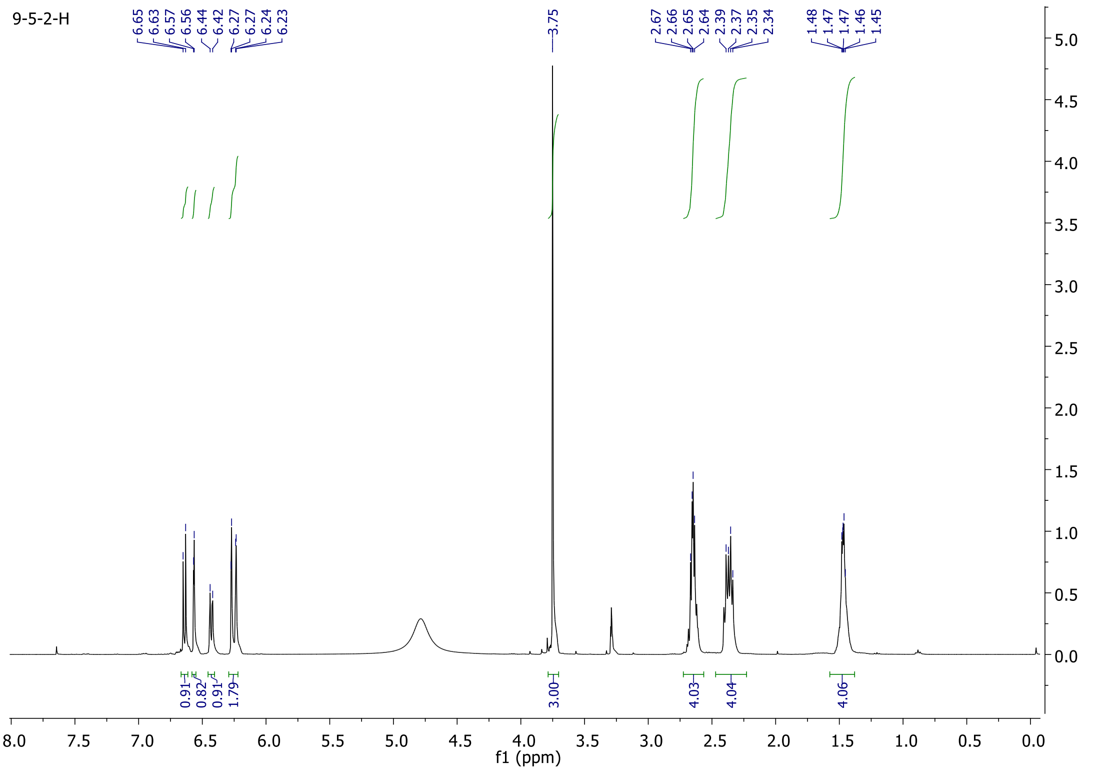
9-5-2-H
6.65
6.63
6.57
6.56
6.44
6.42
6.27
6.27
6.24
6.23
3.75
2.67
2.66
2.65
2.64
2.39
2.37
2.35
2.34
1.48
1.47
1.47
1.46
1.45
0.91
0.82
0.91
1.79
3.00
4.03
4.04
4.06
f1 (ppm)
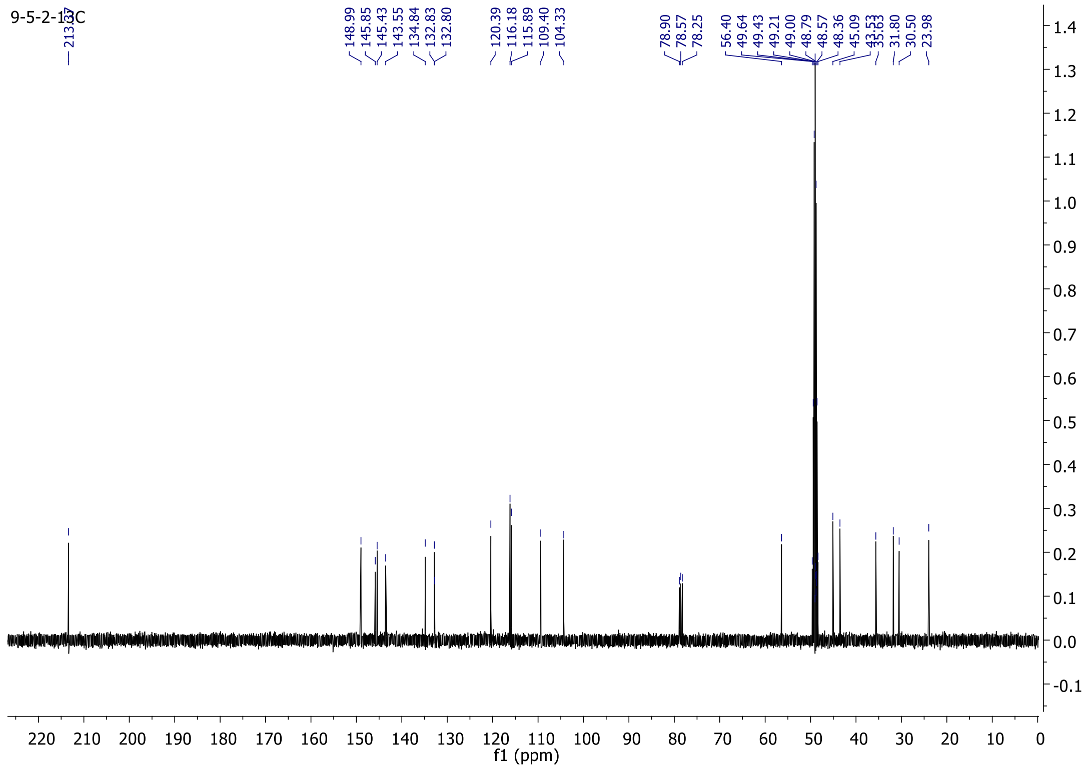
9-5-2-13C
148.99
145.85
145.43
143.55
134.84
132.83
132.80
120.39
116.18
115.89
109.40
104.33
78.90
78.57
78.25
56.40
49.64
49.43
49.21
49.00
48.79
48.57
48.36
45.09
43.53
35.63
31.80
30.50
23.98
f1 (ppm)

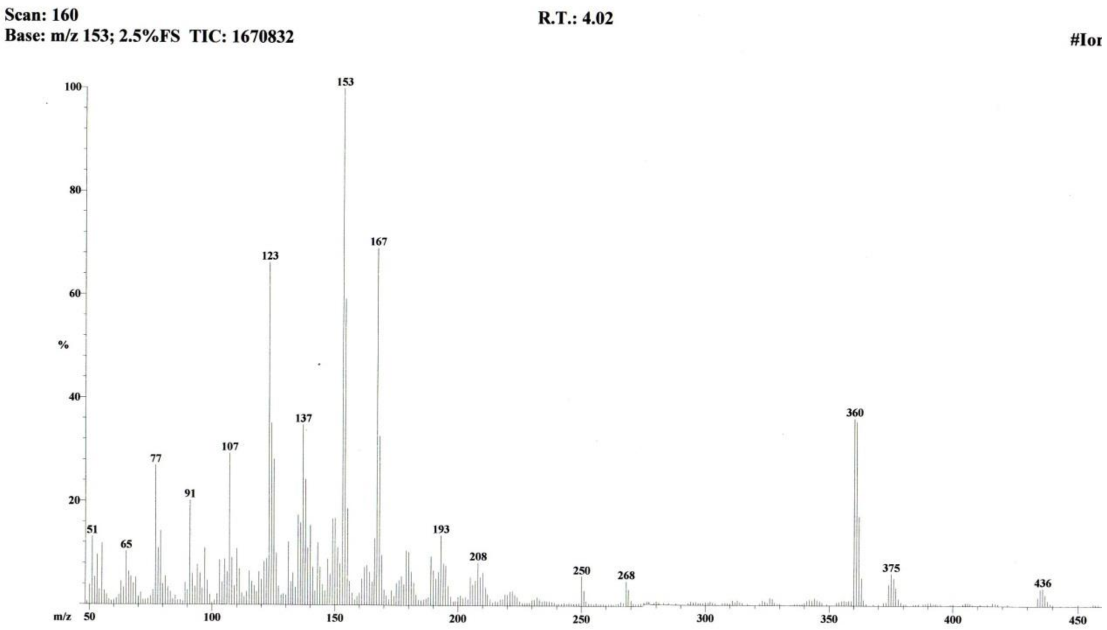

Figure S6. ^{1}H and ^{13} C NMR and Ms charts of **dihydrogingerenone-A (6)**

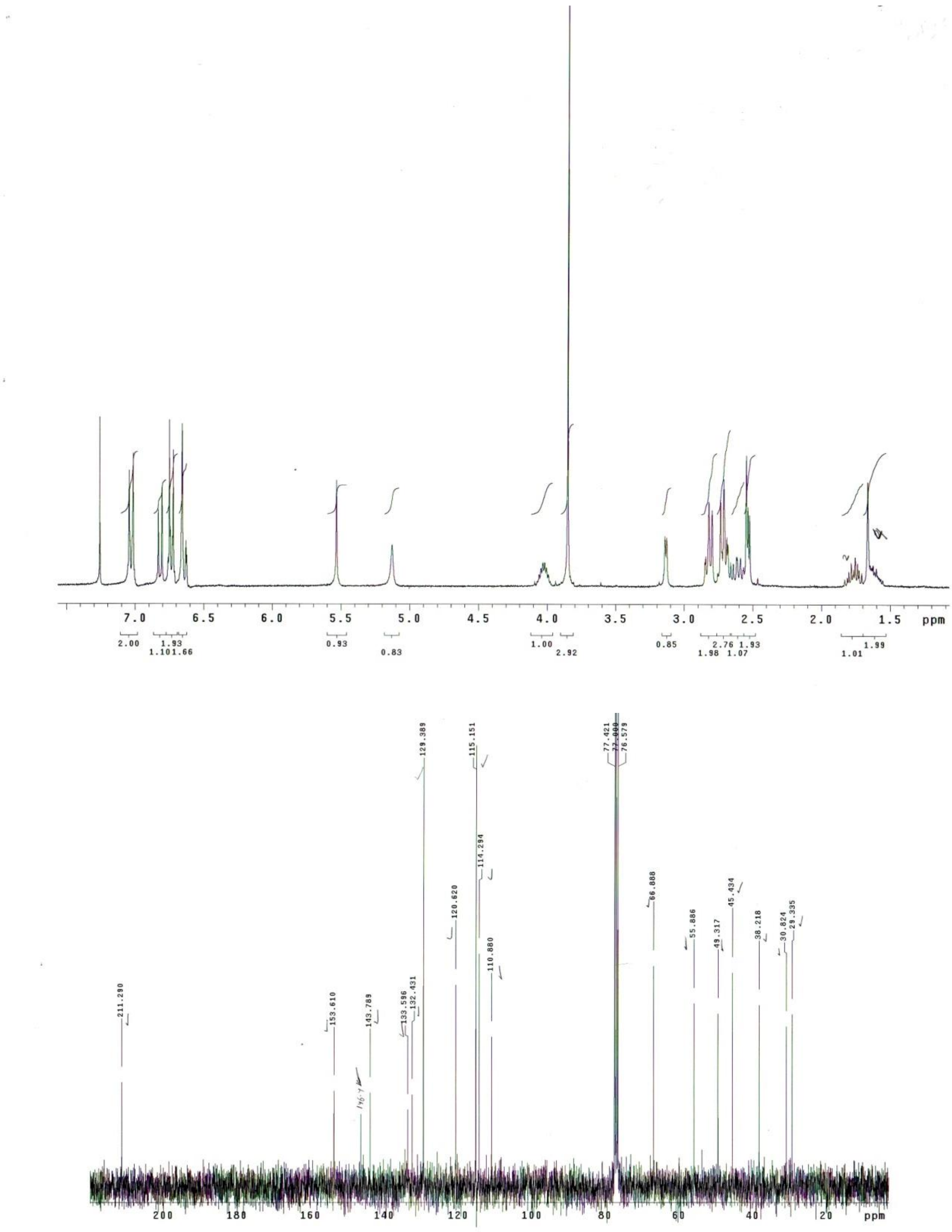

Figure S7. ^{1}H and ^{13} C NMR charts of **dihydrogingerenone-C (7)**

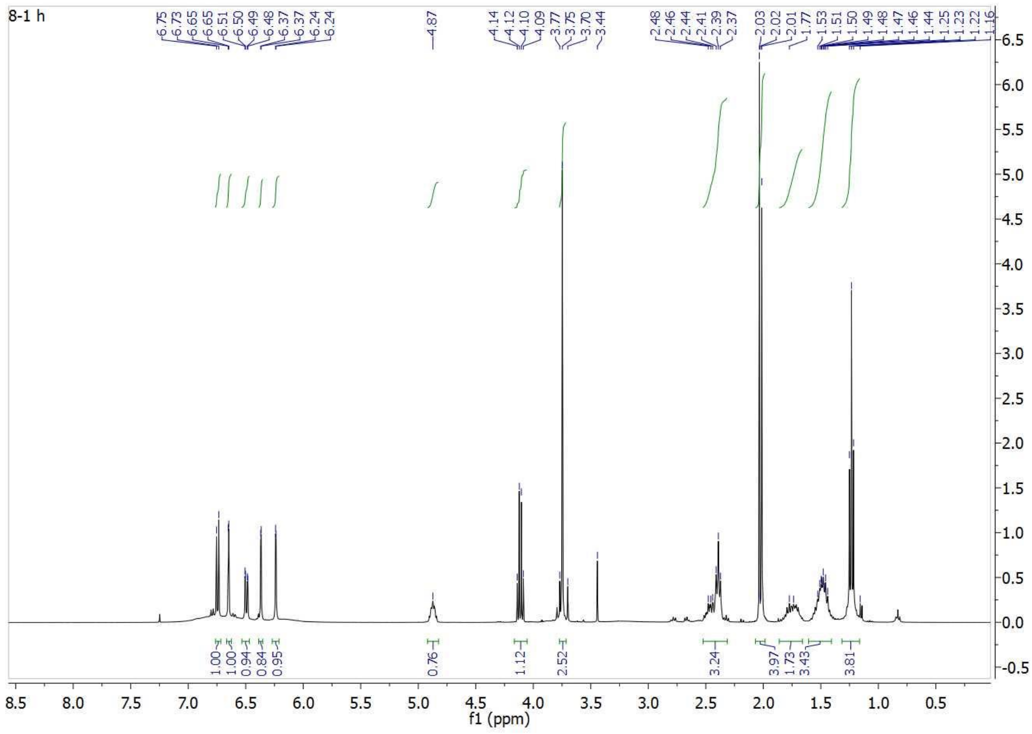

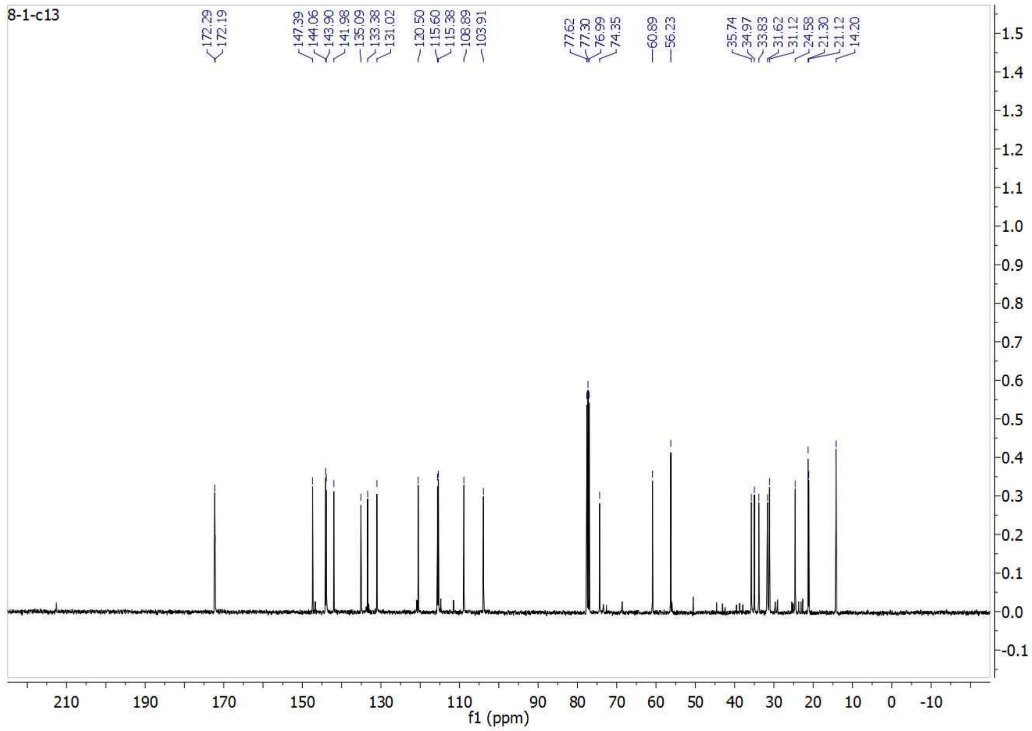

Figure S8. ^{1}H and ^{13}C NMR charts of **compound 8**

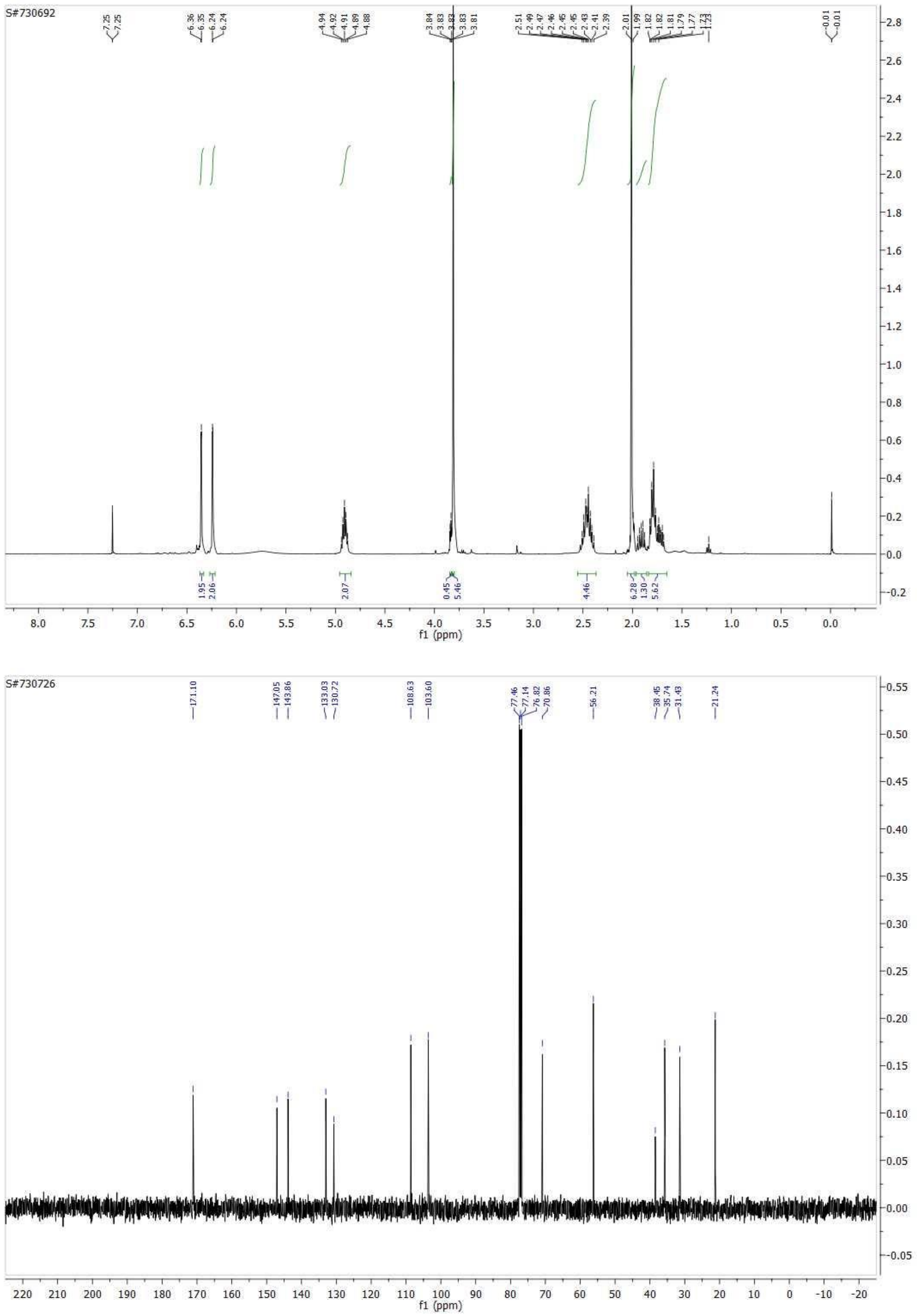

Figure S9. ^{1}H and ^{13}C NMR charts of **compound 9**

Report of Specimen1

Specimen Name: Specimen1 Run Time: 11/8/2016 2:22 PM

Cytometer: NovoCyte 451141011592 Software: NovoExpress 1.1.0

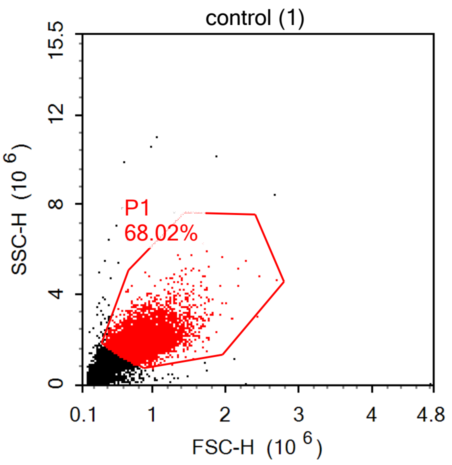

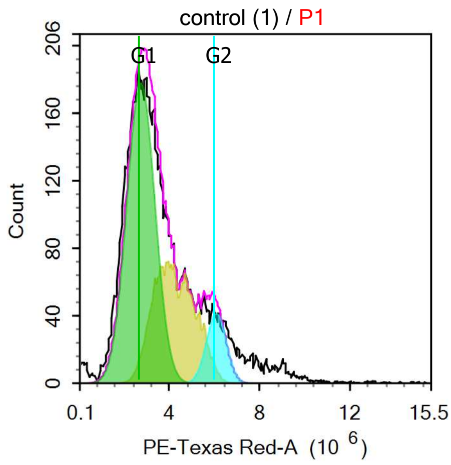

Gate	Count	% All	Mean X	Mean Y
All	12,000	100.00 %	661,934	1,522,012
P1	8,162	68.02 %	814,598	1,906,709

RMS	Freq G1	Freq S	Freq G2	G2/G1	CV G1
11.50	52.54	31.35	8.67	2.21	22.48 %

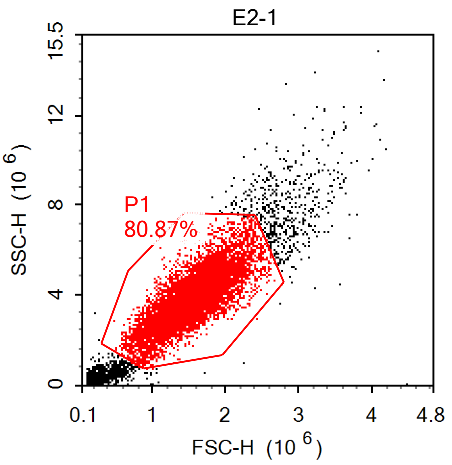

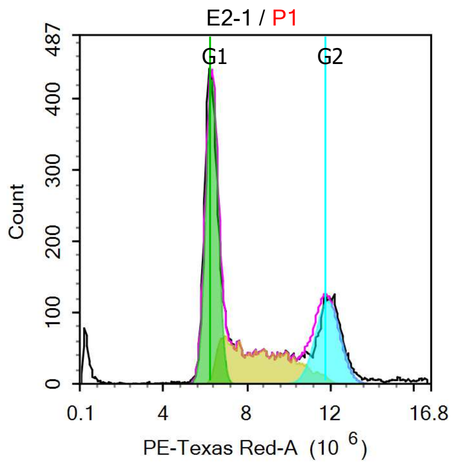

Gate	Count	% All	Mean X	Mean Y
All	12,000	100.00 %	1,356,756	3,379,520
P1	9,704	80.87 %	1,478,921	3,699,377

RMS	Freq G1	Freq S	Freq G2	G2/G1	CV G1
15.72	41.83	29.44	23.25	1.88	4.56 %

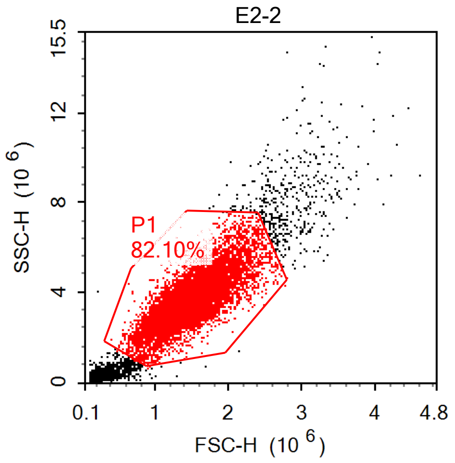

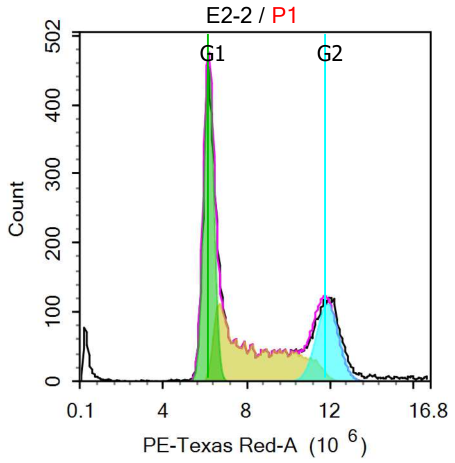

Gate	Count	% All	Mean X	Mean Y
All	12,000	100.00 %	1,362,533	3,343,269
P1	9,852	82.10 %	1,484,045	3,647,778

RMS	Freq G1	Freq S	Freq G2	G2/G1	CV G1
11.04	36.81	36.63	19.89	1.92	3.92 %

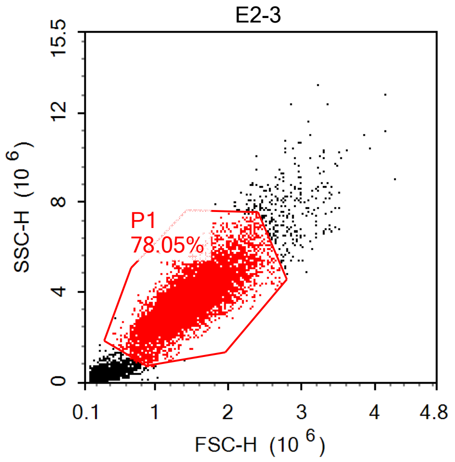

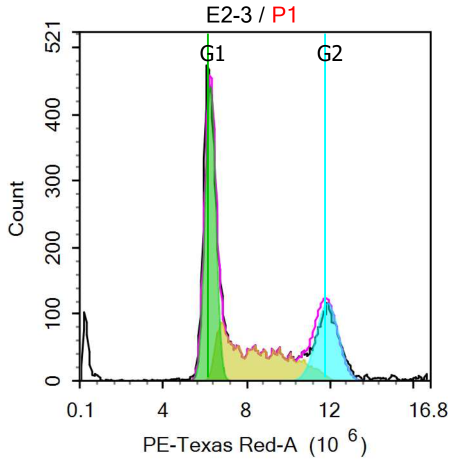

Gate	Count	% All	Mean X	Mean Y
All	12,000	100.00 %	1,244,211	3,028,538
P1	9,366	78.05 %	1,441,499	3,563,016

RMS	Freq G1	Freq S	Freq G2	G2/G1	CV G1
16.50	39.07	32.04	21.73	1.90	4.02 %

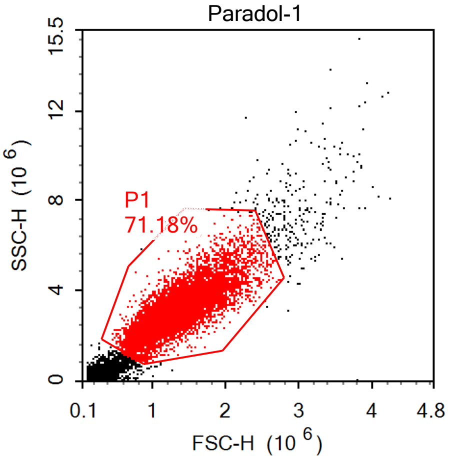

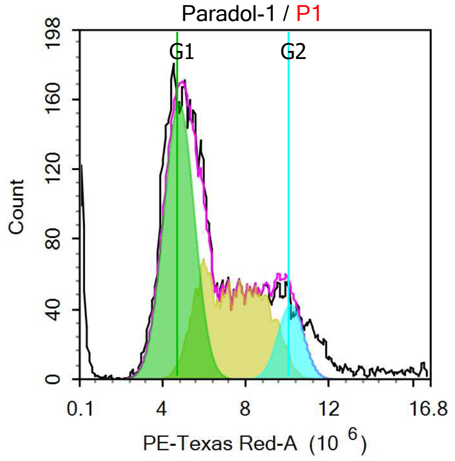

Gate	Count	% All	Mean X	Mean Y
All	12,000	100.00 %	1,099,213	2,490,806
P1	8,542	71.18 %	1,363,014	3,175,923

RMS	Freq G1	Freq S	Freq G2	G2/G1	CV G1
10.77	41.79	34.58	9.56	2.11	15.06 %

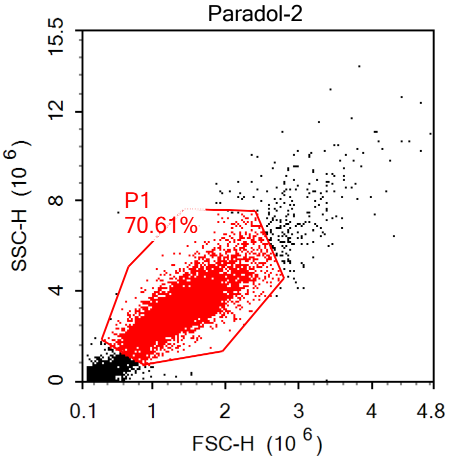

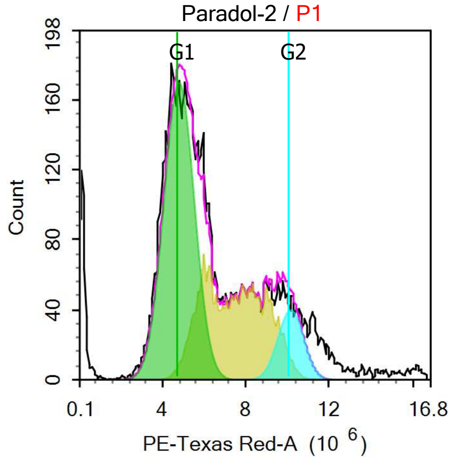

Gate	Count	% All	Mean X	Mean Y
All	12,000	100.00 %	1,094,594	2,489,568
P1	8,473	70.61 %	1,359,290	3,180,320

RMS	Freq G1	Freq S	Freq G2	G2/G1	CV G1
10.63	44.84	33.32	8.93	2.11	15.06 %

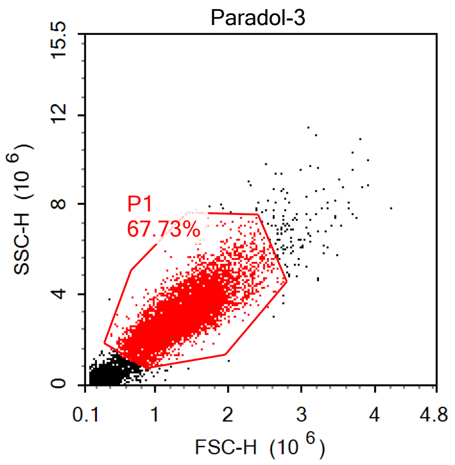

Gate	Count	% All	Mean X	Mean Y
All	12,000	100.00 %	1,007,418	2,248,819
P1	8,127	67.73 %	1,324,895	3,076,992

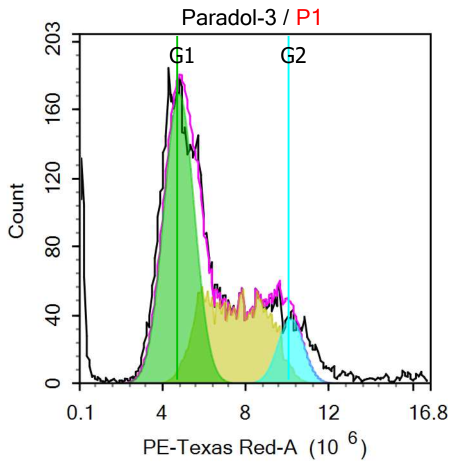

RMS	Freq G1	Freq S	Freq G2	G2/G1	CV G1
11.48	46.88	32.19	8.85	2.11	15.06 %
